# Molecular cloning, GTP recognition mechanism and tissue-specific expression profiling of myxovirus resistance (Mx) protein in *Labeo rohita* (Hamilton) after Poly I:C induction

**DOI:** 10.1038/s41598-019-40323-0

**Published:** 2019-03-08

**Authors:** Basanta Kumar Das, Pragyan Roy, Ajaya Kumar Rout, Deepak Ranjan Sahoo, Soumya Prasad Panda, Sushmita Pattanaik, Budheswar Dehury, Bijay Kumar Behera, Sudhansu Sekhar Mishra

**Affiliations:** 1 Fish Health Management Division, ICAR-Central Institute of Freshwater Aquaculture, Kausalyaganga, Bhubaneswar, 751012 India; 2 Biotechnology Laboratory, ICAR-Central Inland Fisheries Research Institute, Barrackpore, Kolkata, 700120 West Bengal India; 3 Biomedical Informatics Centre, ICMR-Regional Medical Research Centre, Nalco Square, Chandrasekharpur, Bhubaneswar, 751023 Odisha India; 4 Present Address: Department of Chemistry, Technical University of Denmark, DK-2800 Kongens Lyngby, Denmark

## Abstract

The myxovirus resistance (Mx) proteins belong to interferon-induced dynamin GTPase and play pivotal role in the inhibition of replication of numerous viruses. These antiviral proteins are released in usual or diseased condition to prevent the viral attack and to carry regular cellular activities like endocytosis and trafficking of nucleoproteins into the nucleus. The invasion of virus up-regulates the expression of Mx transcripts and double-stranded RNA mimic like polyinosinic polycytidyilic acid (Poly I:C). To understand the tissue-specific expression profiling and mechanism of GTP recognition of Mx protein from *Labeo rohita* (rohu), the full-length gene was cloned, sequenced and characterized through various Bioinformatics tools for the first time. The Mx cDNA was comprised of 2297 bp, and the open reading frame of 1938 bp encodes polypeptide of 631 amino acids. The coding sequence of Mx protein possess the signature motif of dynamin superfamily, LPRG(S/K)GIVTR, the tripartite guanosine-5/triphosphate (GTP)-binding motif (GXXXSGKS/T, DXXG and T/NKXD) and the leucine zipper motifs at the C-terminal end, well conserved in all interferon-induced Mx protein in vertebrates. Western blotting confirmed the molecular weight of Mx protein to be 72 kDa. After the intraperitoneal challenge of *L. rohita* with a Poly I:C, up-regulation of Mx protein was observed in brain, spleen, liver, kidney, intestine, heart, muscle, and gill. Ontogeny study displayed pronounced expression of Mx protein in all stages of the developmental of Rohu after Poly I:C induction. However a persistent expression of Mx transcript was also observed in Rohu egg as well as milt without induction with Poly I:C. Higher expression of Mx gene was observed on 96 h where it was 6.4 folds higher than the control. The computational modelling of Mx protein portrayed the tripartite N-terminal G-domain that binds to GTP, the bundle-signaling element (BSE) which interconnects the G-domain to the elongated stalk domain and C-terminal helical stalk domain. In agreement with the experimental studies, a series of conserved residues viz., Gln52, Ser53, Ser54, Leu68, Pro69, Gly71, Gly73, Thr76, Asp151, Gly154, Thr220, Lys221, Val251, Cys253, Arg254, and Gly255 were computed to be indispensable for tight anchoring of GTP within binding cavity of G-domain. The binding free energy calculation study depicted that the van der Waals and electrostatic terms contributs significantly to molecular recognition of GTP. Collectively, our study provides mechanistic insights into the tissue-specific expression profiling and GTP binding mechanism of Mx protein from *Labeo rohita*, which is expected to drive further research on several cellular events including viral resistance and endocytosis in the near future.

## Introduction

The Innate immune system is the first line of defense system against the invasion of virus’s in vertebrates. This first-line antiviral defense is precisely regulated by interferons (IFNs) and interferon-induced genes through a wide array of cellular mechanisms. In mammalian system, a varied range of cellular pathways is being activated upon infection by viruses leading to the release of nearly 200 interferon-stimulated genes (ISGs)^1^. In most cases, several genes mediate the antiviral response, but sometimes a single gene also regulates it. Type I and Type III IFNs stimulate the expression of several ISGs, which have an antiviral, antiproliferative, and immunomodulatory function. Three most studied enzyme systems that mediate antiviral pathways are protein kinase R (PKR)^2^, the 2–5 OAS/RNaseL system^3^ and the Mx GTPases^4^. The IFN-induced myxovirus resistance (Mx) proteins are powerful antiviral factors, which attenuate replication of a broad range of viruses including influenza viruses in mammals and other vertebrates^5^. The expression of Mx gene depends on induction by type I (alpha or beta) or type III (lambda) IFNs^6^, whereas the action of Mx proteins is IFN independent. In case of humans, two Mx proteins (MxA and MxB), are found to express, and part of the interferon response to viral infection. Through both share high percentage of homology, but they have shown the difference in their antiviral activities, signifying that these closely related proteins have distinct mechanisms of action. For instances, MxA restricts replication of a variety of RNA and DNA viruses whereas, MxB, inhibit HIV-1 replication^7^. The mechanistic basis of Mx-mediated antiviral activity remains mostly indeterminate.

The Mx proteins restrict dissimilar viral families that are genetically and structurally diverse^8^. Mx proteins are large GTPases and belong to the group of IFN-induced GTPases occupied in the control of intracellular pathogens. Owing to their sequence similarity, biochemical, and structural properties, Mx proteins strongly resemble the class of dynamin-like GTPases that mediate basic cellular processes involving membrane remodeling^9^. The dynamin-related proteins (DRPs) are enzymes generally categorized into two categories i.e., those that promote membrane fission (exemplified by dynamin) and those that promote membrane fusion (exemplified by atlastin). Extensive structural and biochemical analysis have revealed that like most dynamin-like GTPases, Mx proteins comprised of conserved N-terminal GTPase (G) domain that binds and hydrolyzes GTP, a central bundle signalling element (BSE) domain which connects and transmits signals between the GTPase and the stalk domains (MD), and C-terminal diverges GTPase effector domain (GED) with leucine zipper motifs (LZ)^10^. The BSE stalk domain is indispensable for oligomerization^10,11^. It has been observed that the GTPase activity and oligomerization are critical for viral inhibition by MxA^12^. Similarly, to dynamin, human MxA assembles into tetramers and displays concentration-dependent oligomerization and assembly-stimulated GTPase activation. The stalk mediates assembly of the enzyme into rings or helical filaments^11^. The tip of the stalk projects into the interior of the assembly and is critical for target recognition. In the Mx proteins, the tip incorporates a flexible and surface exposed loop (the “L4 Loop”)^11^ that is responsible for lipid binding and specific recognition of some viral structures^13,14^. The GTPase domains decorate two opposing faces of the resulting filament or ring. They are connected to the stalk by the BSE, composed of three non-contiguous helices, drawn from the start, middle, and end of the sequence. The role of GTPase domain dimerization in regulating GTP hydrolysis by the DRPs, and the connection with the mechanical activity, were established by several recent structural analyses^15–18^. In most cases, the antiviral activity of Mx proteins is firmly linked to GTP binding or GTP hydrolysis^19,20^.

In recent years, the critical role of Mx proteins in antiviral protection has been established in cell culture and animal model systems. In fishes, the antiviral property of Mx protein has also been reported. Mx has been cloned and characterized in rainbow trout, *Oncorhynchus mykiss* Walbaum^21^; Atlantic salmon *Salmo salar* L^22^; Japanese flounder *Paralichthys olivaceus*^23^; fugu *Takifugu rubripes*^24^; channel catfish, *Ictalurus punctatus*^25^; orange-spotted grouper *Epinephelus coioides*^26^; rare minnow *Gobiocypris rarus*^27^; gilthead sea bream *Sparus aurata*^28^ and grass carp *Ctenopharyngodon idella*. Several works have been done on maternal transfer of immune molecules in different variety of fishes. In unfertilized eggs of brown trout, *Salmo trutta* mRNA transcripts of lysozyme was detected^29^ and in Coho salmon, Onchorynchus kisutch and Chinnok salmon *Onchorynchus tshawytscha* lysozyme activity has been reported^30,31^. Interestingly in rainbow trout, maternal transfer of IgM and complement components has been studied by^32^. The ontogenic study of Mx gene expression at diffrent developmental stages have been reported in C. mrigala^33,34^. In fishes, the Mx gene is induced by Type-I Interferon, the main cytokine mediating the innate immune antiviral function. Although the antiviral nature of human Mx has been investigated, the exact role of teleost Mx protein in preventing viral replication and assisting Type I IFN is not very clear. Therefore, the present study was conducted to explore the function of Mx gene in Rohu by analyzing its activity following the administration of Poly I:C, a synthetic double-stranded RNA which mimics the dsRNA like the structure of Grass Carp Reo Virus (GCRV), Viral Haemorrhagic Septicemia Virus (VHSV) and (SVCV). In this study molecular cloning, *in-silico* characterization of full-length Mx CDS of Rohu and expression kinetics of transcript after Poly I:C induction was performed in different tissues. Studies on the functional aspects of Mx protein in the developmental stages of Rohu will throw light on the various aspects of innate immunity and the role of maternal transfer of immune factors in hatchling immunity. Collectively, our study provides mechanistic insights into the structure-function mechanism and GTP recognition by Mx protein along with signal transduction cascade that leads to the up-regulation Mx protein upon induction by Poly I:C in different tissues in important fish like Rohu.

## Results

### Sequence and domain architecture study

The 3′ and 5′ ends of the Mx gene sequence were obtained through RACE PCR and a full-length Rohu Mx sequence of 2297 bp was obtained (Fig. S1). The predicted ORF of Mx protein (1938bp) encodes a 631 amino acid long polypeptide with a molecular weight of 71.65 kDa (with  an isoelectric point of 8.86). The Mx protein of Rohu displayed maximum sequence identity with that of Mx protein of mrigala (94.4%) followed by *Squaliobarbus* and *Ctenopharyngodon* 83.4%, *Gobiocypris rarus* 83.2%, *Carassius auratus* 82.1%, *Danio rerio* 78.4% (Table S2). Domain architecture through CD search and SMART revealed three domains, i.e., the tripartite GTP binding domain (at the N-terminal end), the BSE domain and GTPase effector domain without the LZ motif (at the C-terminal end). A tripartite guanosine-5′-triphosphate (GTP)-binding motif, GXXXSGKS/T (GDQSSGKS) 35–42 amino acid position, DXXG (DLPG)151–154 aa and T/NKXD (TKPD) 220–223 aa motifs typical to dynamin family were found in Mx protein of Rohu. The signatures LPRG(S/K) GIVTR (LPRGTGIVTR) of the dynamin family positioned between (68–77 aa) sequences were predicted to be common to all other Mx protein sequences. Rohu Mx does not contain a nuclear localization signal (NLS), characterized by a short stretch of positively charged (K/R) residues at the C-terminal end. PSORT II and ScanProsite were used to identify nuclear localization, and it was found that the protein mostly localized in the cytoplasm. No potential glycosylation sites (NXT/S) were observed in Mx protein Rohu. Multiples sequence alignment of Mx from Rohu with other Mx proteins revealed that it was highly similar to other teleost Mx proteins.

### Phylogenetic analysis

**Figure 1:**
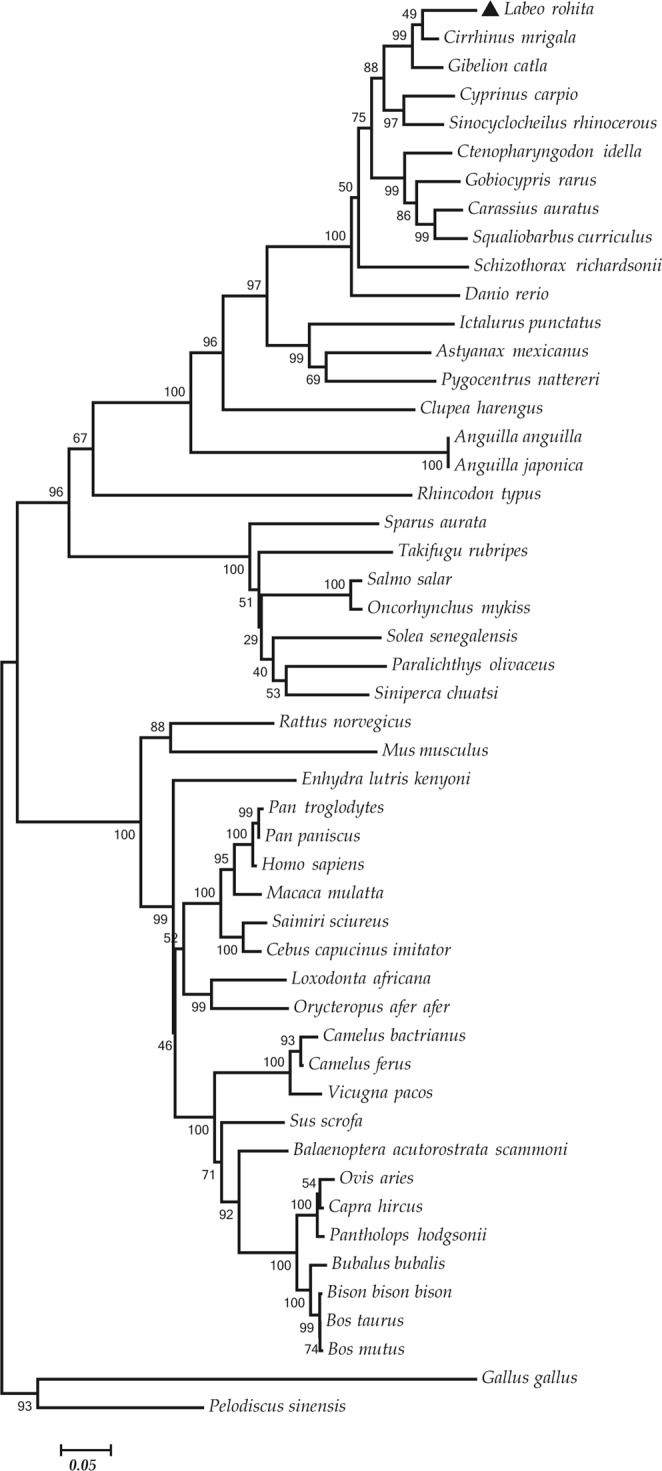
The evolutionary history of Mx_Lr Protein was inferred using Neighbor-Joining method. The optimal tree with the sum of branch length = 5.19236111 is shown. The percentage of replicate trees in which the associated taxa clustered together in the bootstrap test (1000 replicates) was displayed. The evolutionary distances were computed using the Day-Hoff matrix based method and are in the units of the number of amino acid substitutions per site. The analysis involved 50 amino acid sequences. There were a total of 558 positions in the final dataset. Evolutionary analyses were conducted in MEGAv7.0 software Package.

A phylogenetic tree was constructed to study the molecular evolution Mx protein from Rohu with those of homologous sequence of fish, chicken, mouse, human, monkey, goat, rat, pig, sheep, camel, buffalo, turtle, chimpanzee, elephant, and others. A neighbor-joining phylogenetic tree was constructed using the MEGA program with an iteration of 1000 replicates. The phylogenetic tree displayed dichotomy in nature with two distinct clades as shown the (Fig. 1). It can be observed that the teleost Mx proteins diverged from the mammalian Mx. Mx from teleosts formed a single cluster while mammalian and avian formed a different cluster with bootstap value >70%. Rohu was in different clade close to *Ctenopharyngodon idella* and *Gobiocypris rarus*. *Sparus auratus*, *Scophthalmus maximus*, *Solea senegalensis, Paralichthys olivaceus, Takifugu rubripes, Siniperca chuatsi* were, the most distant clade diverged from the major cluster. Rohu Mx was found form a clade with that of *Ctenopharyngodon idella*, and *Gobiocypris rarus*. However, Rohu Mx protein displayed low sequence similarity with that of human, mouse, and chicken, but the GTP binding residues were predicted to fully conserved signifies the conserved evolution of GTP binding mechanism. The computed phylogenetic tree using the GTP binding domain of Mx_*Labeo rohita and its closest homologs* has been depicted in  Fig. S11.

### Tripartite domain architecture of Mx protein

**Figure 2:**
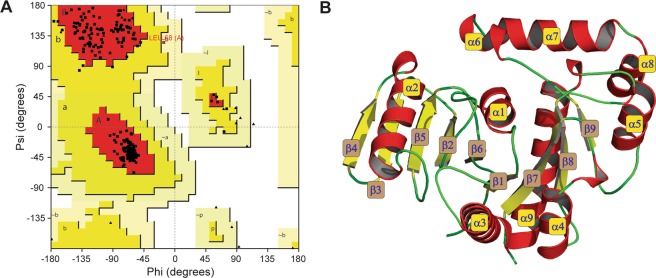
(**A**) Ramachandran plot of the modeled GTPase domain of Mx protein from Labeo rohita. The Plot was generated by PROCHECK Program. (**B**) Three-Dimensional architecture of GTPase domain of Mx protein. The protein has been displayed as solid ribbon representation with α-helices, β-sheets and turns.

The Rohu Mx protein structure (18–620 aa) (Fig. S4A,B) was predicted by theoretical modelling approach multi-template approach. BLASTp search against PDB identified top ranking template structures with percentage identity of 50% i.e., (PDB ID: 3ZYS.B), (PDB ID: 3SZR.A), (PDB ID: 4WHJ.A), and (PDB ID: 5GTM.A) (Fig. S2) with a query coverage of 96%. Based on the multiple-template approach, Modeller facilitated in the development of 200 raw models of which model with least molpdf and the DOPE score were retained for further side-chain and loop refinement using Galaxy loop and Galaxy Refine tools. The refined model was subjected to various model validation servers to assess the stereochemical quality and energy profile of the model (Fig. 2B). Ramachandran plot of the proposed homology models displayed a Phi-Psi distribution of each amino acid of Mx protein. The plot displayed higher the percentage amino acids (94.7%) in the most favoured and allowed region (Fig. 2A) with none of residue had bad geometry (outlier region of Φ and Ψ plot). PSIPRED was employed to derive the secondary structure elements from its primary amino acid sequence (Fig. S3) of the GTP binding domain. The ProSA-web analysis displayed z-score −10.01 (within the range of crystal/NMR structures of the same size) indicating the good quality of the proposed model. In addition, ProQ-Protein quality prediction tool portrayed LG-score of 5.628 seasons, and Max-Sub score of 0.56 indicating the extremely good quality of the constructed Mx model (Table 1). Pair-wise sequence-structure alignment and superposition of the constructed homology models with their respective template structures exhibited a least Cα RMSD value between 0.47–1.32 Å signifying the accuracy of the predicted model. Molecular dynamic simulation offers an approach for refining protein structures, often used for wide spectrum applications including protein design, engineering etc. In this study, the truncated (see materials and methods) portion i.e., only the GTPase domain of Mx protein in Rohu was solvated and subjected to 100 ns MD. From the stable and well-equilibrated trajectory, an average structure was extracted and subjected to Ramachandran plot analysis (as shown in Fig. 2A). Least and stable backbone root mean square deviation (RMSD) of about ~0.25 nm for the modelled GTPase domain indicated the convergence of the modelled structure after MD. After running 100 ns of dynamic simulations, no residue in the outlier region was observed. Further, it was noticed that the number of residues present in the additionally allowed region of Ramachandran plot declined while a number of residues present in favoured region rises significantly and is thus a clear evidence of improved stereo-chemical quality. To further confirm the stability of the GTPase domain, the DSSP algorithm was used to evaluate the changes in a secondary structure during MD simulations. Interestingly no significant changes in structural elements were observed during the entire simulation time (data not shown). A complete detail of model validation has been listed in Table 1.

**Table 1:** Model validation report of GTPase domain of Mx_*Labeo rohita*.

Model Validation Tools	Model validation parameters	Scores (%)
PROCHECK	Most favored region (%)	94.7
	Additional allowed region (%)	4.1
	Generously allowed region (%)	0.4
	Disallowed region (%)	0.8
	Overall G-factor	−0.04
Verify 3D	Averaged 3D-1D Score > 0.2	97.81
ERRAT	Overall Quality (%)	79.69
ProSA	Z-Score	−8.7
ProQ	LG score	5.549
	Max Sub	0.539
MolProbity	Cβ deviations > 0.25 Å	0
	Residues with bad bonds	0
	Residues with bad angles	0.1
METAMQAPII	GDT_TS	45.347
	RMSD	3.542

### Convergence of the simulation systems

**Figure 3:**
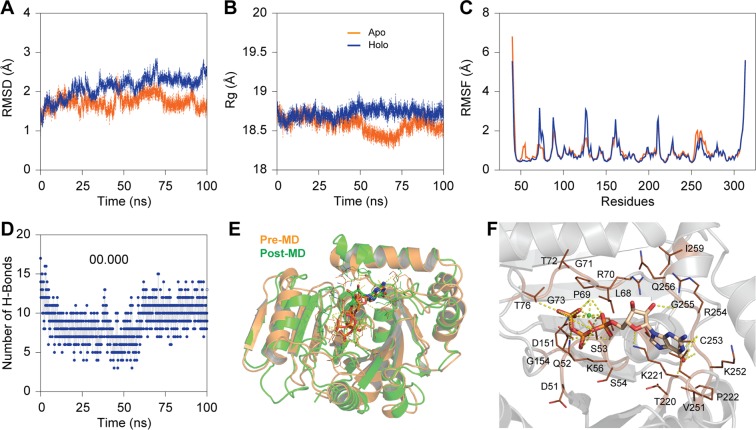
Conformational stability of apo and holo (in presence of Mg + 2) conformers of GTPase domain i.e., Mx Labeo rohita-GTP (manual docked) complexes during 100 ns MD simulation. (**A**) Root mean square deviation (RMSD) of backbone atoms of the modeled of apo and holo conformers. (**B**) Radius of gyration (Rg) plot showing the compactness of trajectory of apo and holo conformers of GTPase domain. (**C**) Comparative analysis of the root mean square fluctuation (RMSF) for Cα atoms of Mx Labeo rohita. (**D**) Variation of H-bonds participated in the intermolecular interaction of GTP with modeled GTPase domain of Mx protein during 100 ns simulation. (**E**) Superimposition of pre‐ and post‐MD GTPase-GTP complex before and after MD simulation. (**F**) Interactions of GTP with the active site amino acids of GTPase domain of Mx protein from Labeo rohita shown in PyMOL (in presence of Mg + 2 ions). The protein, ligand and hydrogen bonds are shown as cartoon, stick and dotted lines, respectively.

RMSD is generally used to access the dynamic stability of systems, as it represents a global measure of protein fluctuations. To assess the stability of MD simulations equilibrium in both and apo form (GTP-bound form) of GTPase domain, the RMSD of the backbone atoms relative to the equivalent native structure as a function of time was calculated. As evident from (Fig. 3A), most of the values of RMSD fluctuate between 1.47 Å and 2.47 Å, and MD simulations of both the systems have reached equilibrium after around 75 ns. Though the GTP-bound system displayed a higher RMSD of ~2.37 Å than that of apo form, both the systems reached equilibrium with a stable RMSD after 70 ns till 100 ns. To determine the desired compactness of both the systems, the Rg was calculated as a function of time during the entire simulations period. Like the RMSD, the apo system was found to be compact with least Rg of ~18.31 Å. Though Rg of the holo system was found be on the higher side, both the systems were found be compact with an average Rg of 18.62 Å (Fig. 3B). Root-mean-square fluctuations (RMSF) of Cα atoms provide direct insight into the structural fluctuation and flexibility of proteins. RMSF values of Cα atoms in the two different conformations of the GTPase domain were computed (as shown in Fig. 3C). Except for the two terminal ends, RMSFs of the loop regions produce higher peaks with most fluctuations ranging from 0.38 Å to 3.12 Å. These changes in RMSF reflect that the flexible nature of GTPase domain of Mx protein. Further, minute observation of the trend in RMSF of GTP-bound system displayed that critical residues aid in GTP binding has higher fluctuation than others. Using the *gmx hbond* utility toolkit in GROMACS, the number of inter-molecular hydrogen bonds and their occupancy during the MD simulations were determined. However, there was a significant decline in number H-bonds during, 25–75 ns, the H-bonds at the docking level were maintained during the MD simulations. Although few critical H-bonds were found to be broken during MD, were later well compensated through new H-bonds and hydrophobic contacts. In case of a GTP-bound system, the average number H-bonds between the GTPase domain and GTP atoms were found to be ~13.17 (Fig. 3D). The observed differences in H-bond formation can be precious for tight anchoring of GTP by GTPase domain of Mx protein and of vital relevance to GTP hydrolysis.

### Principal component analysis

**Figure 4:**
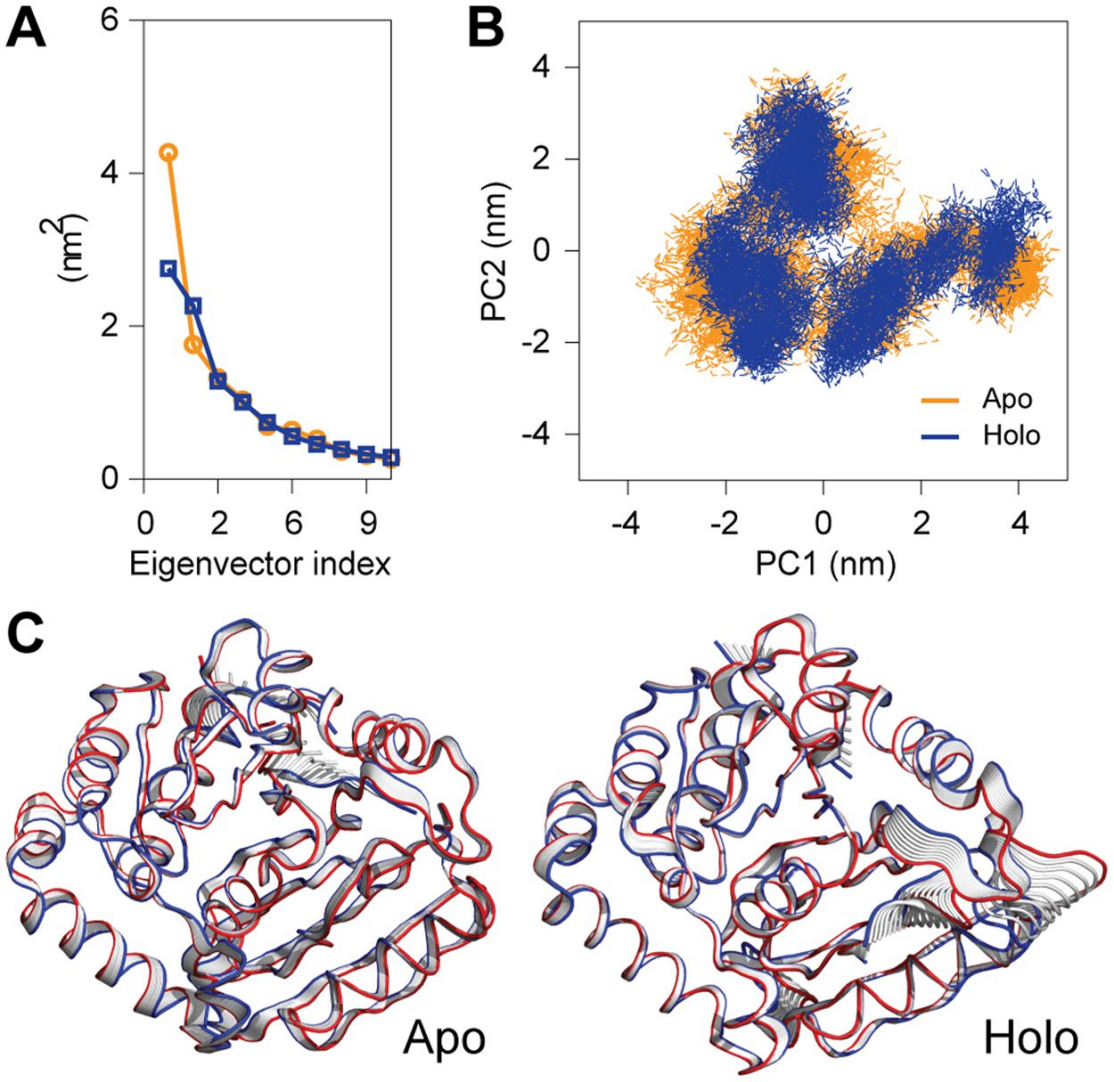
Principal component analysis (PCA) of apo and holo conformers GTPase domain of Mx protein from Labeo rohita PCA was performed using gmx covar and gmx anaeig utility toolkits of GROMACS. (**A**) The eigenvalues plotted against the corresponding eigenvector indices obtained from the Cα covariance matrix constructed from the 100 ns MD trajectory. (**B**) Projection of the motion of the structures of the backbone atoms (apo and holo forms) of Mx_Lr in phase space along the first two principal eigenvectors (EV1 and EV2). (**C**) The superimposition of structural coordinates associated with the principal component 1 (PC1) of apo conformers displaying the global motion. The superimposition of structural coordinates associated with the principal component 1 (PC1) of holo conformers of GTPase domain displaying the global motion. The initial conformations in blue, the final in red and the intermediate ones were displayed in silver.

**Figure 5:**
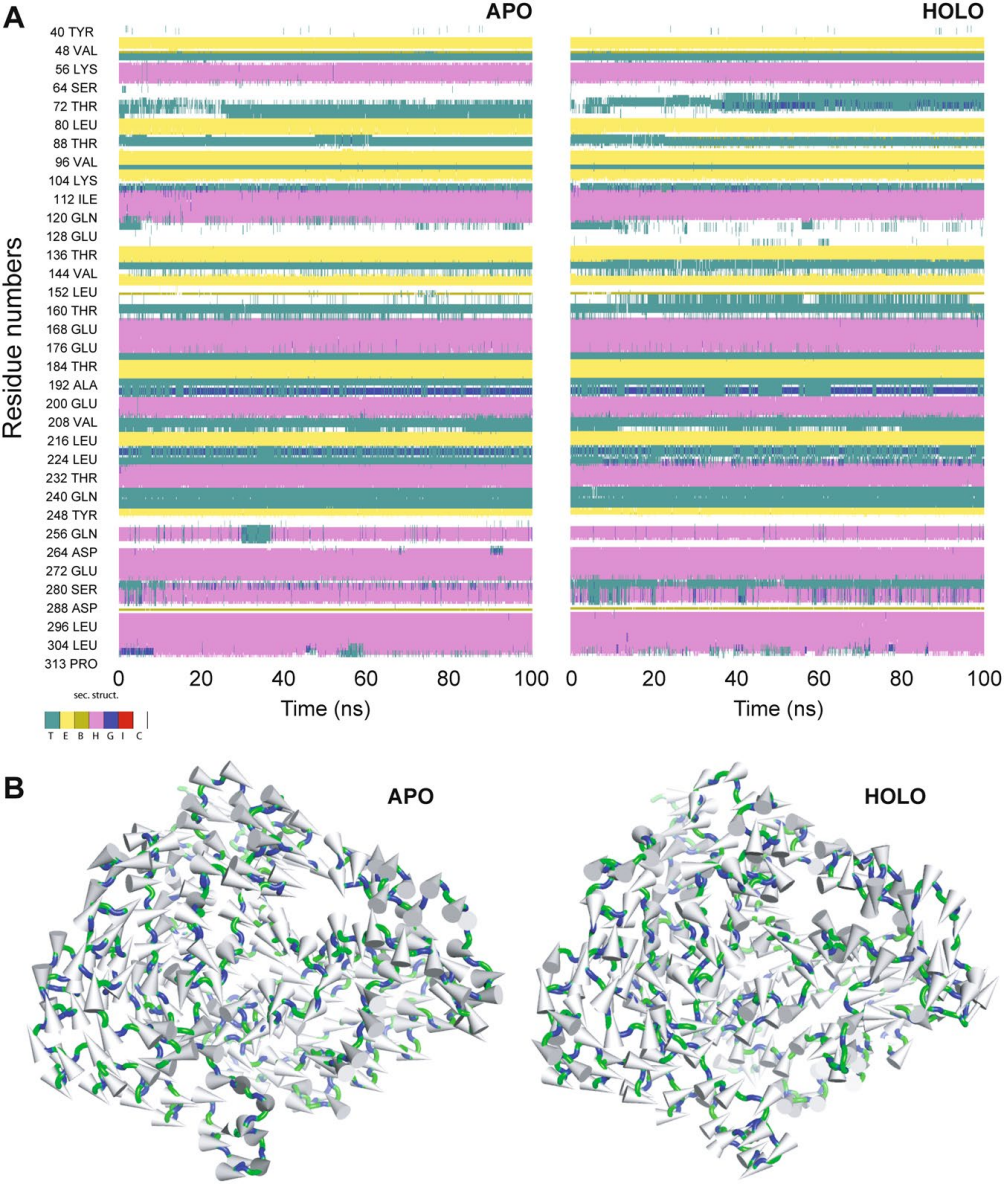
Secondary structure evolution and porcupine plot analysis of apo and holo conformers of GTPase domain. (**A**) Secondary structure components of both Mx_Lr and Mx_Lr-GTP during the course of 100 ns simulation time (the colour segments provided just below the figure represents the secondary structure properties); (**B**) The Porcupine plot of the first eigenvector generated through principal component analysis of the representative structures of GTPase domain and GTPase-GTP complexes. The vectors, represented as arrows, show the tendency of movement.

To understand motion changes of apo and holo conformations of the GTPase domain of Mx protein, PCA was performed on resultant 100 ns MD trajectories. By the diagonalization of the covariance matrix of the Cα atomic fluctuations, the set of eigenvalues thus obtained and plotted with decreasing order versus the corresponding eigenvector indices. The first few eigenvalues corresponding to concerted motions quickly decrease in amplitude to reach some constrained and more localized fluctuation. In this study, the first two eigenvectors (EVs) obtained from capture more than 78.37% and 82.32% of the total motion respectively for the apo and holo form, indicating that these vectors define the essential subspace of the systems. It can be observed that the eigenvalues of apo conformation were higher than the GTP bound conformation (Fig. 4A), which was well supported by the projection of PCs into essential phase space. The scatter plot generated for the apo and holo form GTPase domain as shown in (Fig. 4B) indicated a significant difference between the two systems. The trace of covariance matrix for apo and holo form was computed to be 11.28 nm^2^, and 10.50 nm^2^ respectively, which signifies that apo form showed more dynamic tertiary structural conformations than the holo protein (Fig. 4C). The cross-correlation matrix of the Cα-atom displacement indicated both correlated and anti-correlated motions in the apo system while the holo system indicated mostly anti-correlated motion as shown in (Fig. S5). To quantitatively understand the movement directions captured by the eigenvectors, a porcupine plot was generated using the extreme projections on principal component PC1. The direction of the arrow in each Cα atom represents the direction of motion, while the length of the arrow characterizes the movement strength. The obtained plot suggests that rotational concerted movements were observed in different conformations of the GTPase domain of Mx protein (Fig. 5B). The secondary structure evolution of Mx during 100 ns MD simulations was computed using VMD from the apo and holo trajectory of GTPase domain (Fig. 5A). The important secondary structure elements were found to be stable throughout 100 ns time in aqueous solution.

### Critical residues responsible for GTP recognition

To understand the intermolecular contacts of GTPase domain of Mx protein with GTP, a comparative analysis was performed before and post MD simulation for each complex (Table S1). From the MD trajectory of holo system, the final structure was extracted for protein-ligand interaction studies using PyMOL. Comparative analysis of Pre-MD with that post-MD displayed that MD simulations result in minor changes in orientation ligand (Fig. 3E,F) but, more or less the key residues participate in the GTP recognition were conserved with the Mx protein of Human indicates their role in binding mechanism. A total of 10 conventional H-bonds (apart from 4 carbon H-bonds) were noticed between GTPase domain and GTP with an average inter-molecular distance of ~2.93 Å. GTP consistently bound with the same pose at GTPase domain of Mx protein where Gln52, Ser53, Thr76, Lys221, Cys253, and Arg254 formed a strong network of H-bonds. In addition, Ser57 formed the highest number of 4 H-bonds with GTP indicating its crucial role in ligand recognition mechanism. To further verify, the energetic contributions of the identified residues, a per-residue decomposition of the overall binding energies was performed (as displayed in Supplementary Fig. S6). As evident from the Figure, it can be ascertained that Asp51, Pro69, and Asp151 impaired binding, while Ser54, Lys56, Arg70, 22, Lys221 and Arg254 made significant contributions to the binding of GTP along with few other conserved residues. Apart from H-bonding, some hydrophobic and electrostatic contacts were also observed, where, Lys221 and Cys253 formed two Hydrophobic and electrostatic contacts GTP. Two salt bridges were also noticed in case of NZ atom Lys56 with O1B and O1G atom of GTP respectively. Lys56 also formed two electrostatic contacts with GTP. Overall, the conserved residues through a strong network of H-bond, electrostatic, and hydrophobic contacts tightly anchored GTP within the binding pocket of GTPase domain of Mx Protein.

### Binding free energy

Depending on the nature of the small molecules/ligand, different interactions ranging from H-bonding, hydrophobic, electrostatics and pi-pi interactions exist between ligand atoms and the amino acids presumed to be essential for protein-ligand interaction. All of these interactions contribute to the overall binding free energy. In this study, MM/PBSA approach was used for binding free energy calculation using a numbers snapshot of the protein-ligand complex from MD trajectories. MM/PBSA analysis displayed that polar solvation energies (PSE) impaired binding of GTP to GTPase domain, while, van der Waals (vdW) and electrostatic forces favour the molecular recognition process. The binding energy of GTPase-GTP complex system was computed to be −153.37 ± 1.78 kJ/mol^−1^ where −246.20 ± 0.60 and −1076.41 ± 2.70 kJ/mol^−1^ contributed by van der Waal energy and electrostatic energy respectively (as summarized in Table 2). Interestingly there was a significant increase in the contributions from electrostatic terms, a fact that can be explained by the more number of H-bonds that were formed between the GTPase domain and GTP ligand (Fig. S6).

**Table 2:** MM/PBSA binding free energy of GTP with GTPase domain Mx protein from Rohu.

Energetic terms	van der Waal energy	Electrostatic energy	Polar solvation energy	SASA energy	Binding energy
Energy (kJ/mol)	−246.207 ± 0.596	−1076.413 ± 2.704	1343.902 ± 2.576	−20.881 ± 0.027	−153.371 ± 1.784

The energetic terms were derived using 500 snapshots from the equilibrated portion of trajectory (70–100 ns).

### Mx gene expression in Rohu eggs, milt, twitching, and hatchling stages

The fertilized eggs were transparent and seen floating in clusters. The transparent fertilized egg with the developing embryo could be seen under the microscope. In the twitching stage, developing embryo could be seen moving in the yolksac followed by the hatchling stage. RNA extraction followed by cDNA synthesis and PCR was done to study the expression of Mx transcripts in Rohu eggs, milt, twitching, and hatchling (H) stages at 24 h, 48 h, 72 h and 96 h (H24, H48, H72, H96). Treated egg and treated hatchling stages at 24 h, 48 h, 72 h and 96 h (T H24, T H48, T H72 and T H96 showed higher expression of Mx transcript compared to the untreated egg and twitching stages. To avoid variation in data these samples were analyzed in triplicates by qRT-PCR and the mean value was considered.

Expression of Mx transcript was studied in different developmental stages up to the hatchling stages. Mx could be detected in both the egg and milt, however; its expression was higher in egg compared to the milt and different hatchling stages. Transcript level was highest in the egg and it gradually increased in twitching stages up to H96. When Poly I:C treated egg, treated twitching stages and treated hatchling stages were studied for Mx expression an increase in Mx expression was seen in treated twitching and treated hatchling stages and maximum at 96 h. Using the 2 − ΔΔCT method, the fold change in Mx expression was calculated normalized to housekeeping reference β-actin gene (Fig. S7). A statistically significant up-regulation of Mx transcript was seen in the above-mentioned samples.

### Expression of Mx transcript in protein profile of egg, milt and various twitching stages and immunostaining

Protein sample of egg, milt, treated and control twitching stage, treated and control hatchling stages was run in 10% SDS PAGE. About 20 µg of protein sample was run in each well. In case of a treated twitching stage, treated hatchling several prominent bands were seen out of which a band at 72 kDa was common in eggs and treated twitching and hatchling stages. However, a faint band was also seen in control twitching, control hatchling stage, and milt at 72 kDa.

Protein electrophoreses of the lysates were done on 10% SDS PAGE and polyclonal antibody developed in rabbit was used to check the expression of Mx transcript in egg, milt and various twitching and hatchling stage up to 96 h. Poly I:C treated twitching stages protein profiles were also studied to see the increase in Mx expression as compared to the untreated twitching stages. From the blotting experiment (Fig. S8) it could be seen that expression of Mx transcript was more in the egg. Secondly, expression of the Mx transcript in the treated twitching stages was slightly higher as compared to the control ones. A prominent band was stained at 72 kDa.

### Expression of Mx gene in Rohu fry for a period of 28days

A gradual up-regulation of Mx transcript was seen for the experimental period of 28 days. Mx/β actin ratio for the control group treated with PBS was in the range of (0.3–0.5) for the total experimental period of 28 days. Poly I:C treated fry five in numbers and in replicates was analyzed for constitutive up-regulation of Mx transcript. The 2 − ΔΔCT method was used to calculate fold change in Mx expression normalized to housekeeping reference β-actin gene. On day 1, fold increase in Mx expression relative to β actin was 0.8 fold, on day 2, it was 2 fold increase, day 3 it was 3.4 fold increase, on day 7 it was maximum of 6.4 fold and then a gradual decrease was seen on day 11, day 14 and by day 21 it was 1.5 and day 28 it was 1 fold (Fig. S9). A statistically significant up-regulation of Mx transcript was seen in the above-mentioned samples.

### Protein profiling and staining of Mx transcript in the fry

About 25 µg of tissue protein was electrophoresed in 10% SDS PAGE and immunostaining was done using polyclonal rabbit antibody. Constitutive up-regulation of Mx transcript was seen from day 1 and a prominent immunostained band at 72 kDa was seen on day 5 and day 7 that gradually decrease in intensity which was almost negligible on day 21 and day 28 (Fig. S10).

A fish infected with diseases will be immunologically strong so that the immune genes are transferred to the larvae. To sustain in the pathogenically hostile environment, healthy and immunologically active larvae are protected by several immune factors which are maternally inherited. Earlier workers have reported the maternal transfer of cytokines and its role in the development of a foetus. However, there is no such report on the ontogeny of the immune system of Indian major carps and except a few on the organogenesis of immunocompetent organs and some innate molecules like Complement component C3^35^, IgZ^36^, lysozyme C, lysozyme G, beta-2 microglobulin, toll-like receptor 22-like and transferring^37^. This research work reports for the first time the ontogenic profile of Mx transcript in the different developmental stages of Rohu that is an egg, twitching stage, hatchling stage, and fry.

### Semi-quantitative RT-PCR

**Figure 6:**
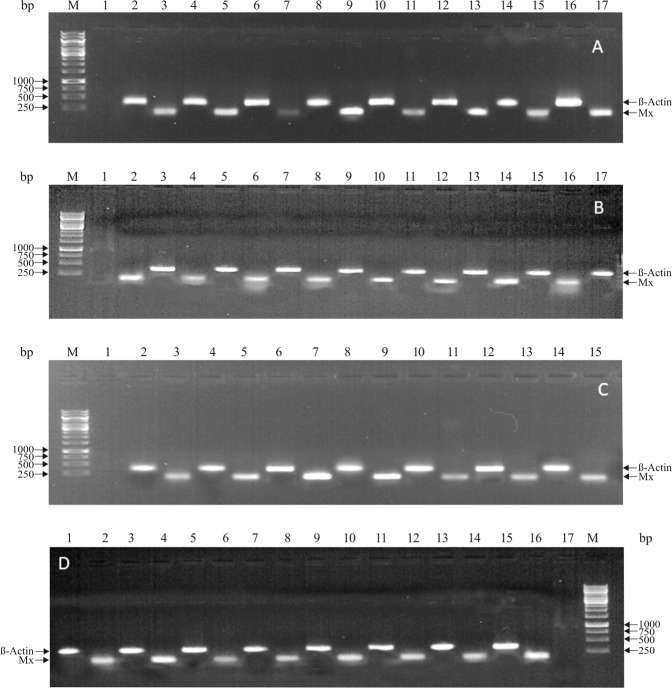
(**A**–**D**) Expression of β-actin and Mx transcripts in (**A**) Kidney (**B**) Liver (**C**) Muscle (**D**) Spleen after Semi-quantitative PCR. (**A**) Kidney M: Marker; Lane 2; β-actin, Lane 3: Mx (1 day); Lane 4: β-actin, Lane 5: Mx (2 days); Lane 6: β-actin, Lane 7: Mx (3 days); Lane 8: β-actin, Lane 9: Mx (4 days); Lane 10: β-actin, Lane 11: Mx (5 days); Lane 12: β- actin, Lane 13: Mx (7 days); Lane 14: β-actin, Lane 15/17: Mx (14 days). (**B**) Liver M: Marker; Lane1: β-actin, Lane 2: Mx (1 day); Lane 3: β-actin, Lane 4: Mx (2 days); Lane 5: β-actin, Lane 6: Mx (3 days); Lane 7: β-actin, Lane 8: Mx (4 days); Lane 9: β-actin, Lane 10: Mx (5 days); Lane 11: β-actin, Lane 12: Mx (7 days); Lane13: β-actin, Lane 14: Mx (8 days); Lane 15/17: β-actin, Lane 16: Mx (14 days). (**C**) Muscle M: Marker; Lane 2; β-actin, Lane 3: Mx (1 day); Lane 4: β-actin, Lane 5: Mx (2 days); Lane 6: β-actin, Lane 7: Mx (3 days); Lane 8: β-actin, Lane 9: Mx (4 days); Lane 10: β-actin, Lane 11: Mx (5 days); Lane 12: β-actin, Lane 13: Mx (7 days); Lane 14: β-actin, Lane 15: Mx (14 days). (**D**) Spleen M: Marker; Lane1: β-actin, Lane 2: Mx (1 day); Lane 3: β-actin, Lane 4: Mx (2 days); Lane 5: β-actin, Lane 6: Mx (3 days); Lane 7: β-actin, Lane 8: Mx (4 days); Lane 9: β-actin, Lane 10: Mx (5 days); Lane 11: β-actin, Lane 12: Mx (7 days); Lane13/15: β-actin, Lane 14/16: Mx (14 days).

A semi-quantitative PCR was performed to detect the expression of Mx in liver, spleen, kidney, and muscle of Poly I:C induced and healthy fish from day 1 to day 14. The liver, spleen, and kidney showed higher expression level on day 3 and 4. In case of liver tissue, the Mx expression was pronounced up to day 14. For muscle tissue, expression was up-regulated on day 4 and then gradually decreased. PBS injected control fish also showed slight of Mx transcript in the entire experimental period of 14 days. Real-time PCR results of the expression level of Mx transcripts in different tissues were identical to that of results of semi-quantitative PCR (Fig. 6A–D).

### RT-PCR and expression study after induction of Poly I:C

**Figure 7:**
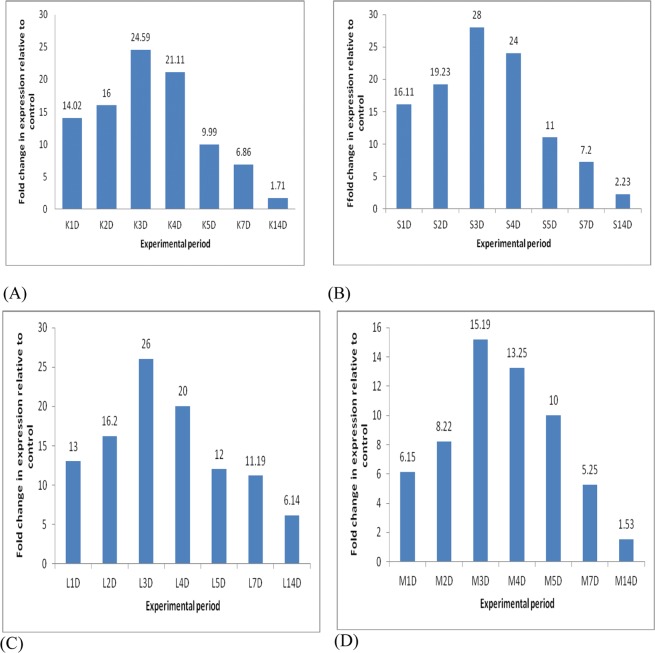
Expression of Mx in (**A**) Kidney, (**B**) Spleen, (**C**) Liver and (**D**) Muscle after Poly I:C stimulation. The relative expression of Mx gene was normalized to the expression of β-actin (internal control) and expressed as fold changes relative to the untreated control group. The mean value of four fish (n = 4) is shown and the bars indicate standard error.

A quantitative RT-PCR assay was designed to study the Mx expression level in different tissues after Poly I:C induction. Fish injected with Poly I:C was studied for the entire experimental period and treated as a positive control and the PBS injected fish as the basal control. Kidney and spleen tissue were chosen for this study because it is a target organ of viral infection. Induction of Mx in the positive control group in spleen (Poly I:C-injected) induced slight Mx expression at day 1 p.i. (2.5-fold) and peaked at day 3 (28), day 4 (24) and then decreased at day 7 (18.7-fold), almost negligible at day 14. Mx was significantly increased in kidney tissue on day 3 (24.59) and day 4 (21.11) and a high level of expression was conserved up to day 7 and then gradually decreased up to day 14. The Similar pattern of Mx expression was observed also for liver tissues. Least expression Mx transcript was observed in the muscle tissue, and the expression pattern on day 1 (6.15), day 2 (8.22), peaked on day 3 (15.19) and gradually decreased up to day 14 (Fig. 7).

### Induction of Rohu Mx protein expression *in vivo* by Poly I:C

**Figure 8:**
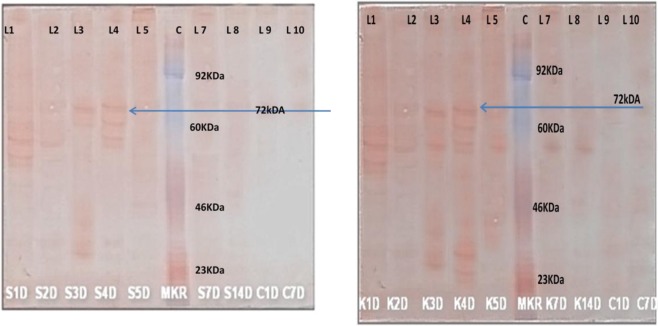
(**A**) Expression of Mx protein in spleen tissue during the 14 day experimental period. The blue arrow shows the expression of 72 kDa Mx protein. (L-1 spleen treated 1 Day, L-2 spleen treated 2 Day, L-3 spleen treated 3 Day, L-4 spleen treated 4 Day, L-5 spleen treated 5 Day, L-6 marker, L-7 spleen treated 7 Day, L-8 spleen treated 14 Day, L-9 spleen control 1 Day, L-10 spleen control 7 Days). (**B**) Expression of Mx protein in kidney tissue during the 14 day experimental period. The blue arrow shows the expression of 72 kDa Mx protein. (L-1 kidney treated 1 Day, L-2 kidney treated 2 Day, L-3 kidney treated 3 Day, L-4 kidney treated 4 Day, L-5 kidney treated 5 Day, L-6 marker, L-7 kidney treated 7 Day, L-8 kidney treated 14 Day, L-9 kidney control 1 Day, L-10 kidney control 14 Days).

The Mx protein expression was studied in different organs of Rohu after stimulation with Poly I:C (Fig. 7). Western blot analysis of Mx proteins in organs like liver, spleen, kidney, and muscle was performed after induction with Poly I:C for a period of 14 days. A strong staining of the putative 72 kDa Mx protein band was observed from day 2 to day 14 after injection of Poly I:C, however, no band was seen in non-treated fish and a faint staining of 72 kDa was seen on day 1 in spleen (Fig. 8A). Maximum expression of Mx protein was seen on day 3–4 after injection of Poly I:C in kidney tissue and relatively high up to the 14^th^ day of the study period (Fig. 8B). Protein bands of lower and higher molecular weights were also observed in the immunoblots of the organ extracts of Poly I:C treated fish. Strong expression of the Mx protein expression in Rohu was seen in spleen, kidney, liver up to 4 days then a gradual decrease in expression was seen up to 14 days. Over the entire experimental period, the expression of putative Rohu Mx protein was found to be lowest in heart and brain. The 72 kDa Mx protein was the major protein band that was detected by the anti-rabbit Mx antibody, other protein bands also were seen and the band pattern was not identical for all the organs.

## Discussion

In this study, the full-length Mx gene of Rohu was cloned and sequenced, which displayed higher level of sequence homology with other Mx genes of mammals and eukaryotes, but significant homology was with Mx genes of teleosts. Like human Mx protein, the dynamin family signature, the GTPase binding site and the leucine zipper at the C-terminal end, characterized the Mx protein of Rohu. Mx proteins possess a highly conserved GTPase domain at the N-terminal end, middle domain or central interactive domain and a GTPase effector domain (GED) including two leucine zippers that form amphipathic helices at the C-terminal end. The typical pleckstrin homology domain (essential for membrane targeting) and proline-rich domain involved in protein-protein interaction specific to members of the dynamin family were absent in Mx proteins^38^. The most conserved domain of Mx protein and dynamin family is the GTPase domain that comprises of a dynamin signature (LPRXXGXXTR) and three GTP-binding motifs (GDXXSGKS, DLPG, and TKPD)^39^. GTPase activity of Mx protein requires Mg^2+^ ion that bind to residues of dynamin signature^40^. The dynamin signature residues also play a vital role in cellular transport processes^11^. The GDXXSGKS, DLPG GTP-binding motifs, which border the dynamin signature, bind the phosphate moiety of GTP, and TKPD GTP binding motif essential for binding guanosine^41^. Similar to other teleost Mx proteins, Rohu Mx protein had the tripartite GTP binding motif and the dynamin signature. The antiviral activity of Mx depends upon GTP binding motif and any mutations in the GTP binding motif presumed to reduce antiviral activity^42^. Oligomerization of Mx protein and virus target recognition requires the middle domain^43^ and the GED domain assists in intra-molecular activation of GTP and the C-terminal leucine zipper of 65 to 70 amino acids in the GED folds back to join the N-terminal GTP-binding domain, forms a functional centre of Mx proteins^23^. The C-terminal leucine zipper was found to be evolutionarily conserved in all Mx proteins suggesting its crucial role in the function of Mx protein. The diverse activities including endocytosis, intracellular vesicle transport and mitochondria distribution, performed by the members of this family depend upon their cellular locations. Several reports substantiate the fact that the antiviral nature of Mx proteins depends upon their cellular location and the type of virus it interacts^4^. PsortII has predicted the probable location of Rohu Mx to be cytoplasmic, which perfectly corroborate with the results of NLS and NES mapper and PROSITE program. Unlike, Rohu Mx nuclear localization signal was found in channel catfish, murine Mx1, Atlantic halibut, rainbow trout Mx2. Like that of sea bream^28^, Atlantic halibut^24^, Japanese flounder^44^, Chinese perch and pufferfish^45^ Mx protein, the probable N-glycosylation sites were not found in Rohu Mx. Though, three potential glycosylation sites were present in the rainbow trout Mx1 and Mx3 sequences^21^, Atlantic salmon Mx1, Mx2 and Mx3^23^, channel catfish Mx sequence^26^ and one in Zebrafish and goldfish. The translation start sequence of Rohu Mx, GTTCAGAC, does not conform to the consensus start sequence, ACCATGG, as determined by Kozak^44^. It has been reported that mammalian Mx also does not have the eukaryotic translational start sequence. Rohu Mx cDNA shows the eukaryotic polyadenylation signal of AATAAA, which was also present in Rainbow trout Mx^22^; Orange-spotted grouper^45^, Red-spotted grouper^46^, Japanese flounder^24^ and all the isoforms of Grass carp^47^, however absent in sea bream^48^. Rohu Mx was identical to a partial sequence of *Catla* Mx protein, followed by Grass carp and Squaliobarbus as all are members of the carp family.

The modelled Mx protein appeared to be an elongated molecule with a three-domain architecture comprised of GTPase domain, bundle-signalling element and the stalk located at the opposing end of the molecule. The N-terminal GTPase domain interconnected with α1 of BSE and stalk through α2 through Proline residue. Previous studies have suggested that the conserved Proline residue act as a hinge mediate conformational coupling of GTPase domain and stalk^16,49^. The stalk was comprised of four helical bundles which superpose well with that human MxA^10,11^. Like that of human MxA, the BSE was composed of three helices surrounded by hydrophobic networks positioned in the centre of Mx molecule. In addition, BSE was found to be connected with the stalk through hinge region (hinge 1) comprised of two loops. Further, it has been reported that mutation of Arg640 in the hinge region leads to reduced GTPase activity and increased endocytosis efficiency^50^. Mutation data of MxA from human suggested that E632A and R640A in the hinge1 region affect the native assembly of MxA oligomeric forms^10^. It has also been reported that mutation of Glu645 (positioned in the α3-helix of BSE domain) to Arg645 results in alteration of antiviral specificity of Mx in Human. The residue was found to superpose well with MxA of human, hence, it can be speculated that the residue might be playing a vital role in the antiviral activity. In case of human, the MxA monomers assemble via the stalks in criss-cross manner to form stalk filament and the self-assembly remains to be crucial for antiviral activity^11^. The MxA oligomer was found to be stabilized by stalk-stalk interactions along with contacts between the stalk and the BSE of neighbouring molecules.

In recent years MD simulation studies have been pivotal to understand the dynamics, intrinsic stability and complex molecular recognition process^51^. In this study, coordinate transfer technique (which performs better than automated docking) and long-term MD was employed to investigate the probable mode of GTP binding by GTPase domain, the results we got promising which perfectly correlate with the experimental data^52^. MD simulations offer a promising approach to understand macromolecular structure-to-function relationships. Accurate use of force fields, scalable hardware along with novel algorithms employed in most MD tools can be of immense importance while addressing complex bimolecular recognition mechanisms. MD simulation study displayed that the conserved residues i.e., Thr76, Pro69, Gly73, Asp151, Gly154, Gln52, Ser54, Gly255, Lys221, Cys253, Thr220, Arg254, Ser53, Gly71, Leu68, Val251 were computed to be essential for GTP binding process which was substantiated by contribution of per-residue decomposition binding free energy analysis. The RMSF analysis also suggested that the GTP binding residues display more fluctuation indicating the active participation in the binding process. Intermolecular H-bond analysis displayed that most of H-bonds retained their active participation in the GTP binding process during MD simulation. PCA suggested that apo form occupies more conformational space than that of GTP bound form, which was well supported by the analysis of stability of MD simulation study. To get an insight into the molecular interactions between GTP with GTPase domain, binding free energy for the complex from MD trajectories was evaluated using MM‐PBSA method. This method has been extensively employed in a number of studies as an efficient and reliable free energy simulation method to understand the model molecular recognition mechanisms^53^. MM/PBSA analysis portrayed that apolar salvation energy contributes significantly to the binding of GTP where van der Waals and electrostatic interaction were the major driving force behind the recognition. The energetic contributions were well supported by the intermolecular contact analysis where the number of H-bonds and electrostatic contacts were found to be dominating among other interaction types. The Mx mRNA expression in Rohu was quantified after Poly I:C induction. Constitutive Mx expression was studied in the brain, heart, spleen, kidney, liver, intestine, muscle, gill and blood and higher expression was seen in spleen, liver, kidney and muscle as compared to other tissues during entire 14 days experimental period. The up-regulation of Mx expression after Poly I:C induction was more pronounced on 3^rd^ and 4^th^ day post-induction compared to control fish. Up-regulation of Mx expression in different tissues after Poly I:C stimulation has already been studied in other teleost species suggesting a role of Mx in viral defence. In trout after Poly I:C induction Mx mRNA was detected in fish by Northern Blot Analysis. Trobidge and Leong^22^ observed Mx mRNA expression after 72 h of treatment of rainbow trout gonad cells with dsRNA. However, Staheli *et al*.^47^ detected Mx mRNA after 24 hr of induction that peaked at 48 hrs. Constitutive expression of Mx was found in kidney, spleen, thymus, intestine, liver, brain, gill, muscle in *E. akaara*^46^. In case of trout and eel^54^ Mx expressions of different isoforms have been studied after Poly I:C induction. Each isoform increased significantly at mRNA level in almost all organs following Poly I:C stimulation in the intestine (about 510 folds) and muscle (about 211 folds) at 6 hrs post infection. In *Sparus aurata* Poly I:C significantly up-regulated expression of Mx in the muscle, liver, head kidney, spleen, brain, heart and gills. Similarly, in sea bream, up-regulated expression was observed in liver, brain, spleen, gills, and muscle after 24 h of injection^28^. Channel Catfish ovary cells were found to express Mx transcript in response to Poly I:C stimulation^25^. After transfection of CHSE-214 cells with Poly I:C, cells produced IFN, suggesting that the previous lack of response to Poly I:C might be due to defect in its uptake^55^. Rodriguez and Perez^56^, reported that fishes inoculated with Poly I:C showed an up-regulated Mx expression levels in the first-day post- infection, with decreased levels at second day and almost negligible levels at 7-day post infection perhaps Poly I:C effect is not pronounced during viral infection. Similarly, in sea bream Mx expression was stimulated after Poly I:C induction both *in vitro* in head kidney macrophages and blood leukocytes and *in vivo* in liver, spleen, head kidney and muscle signifying the role of Mx in the antiviral response.

The expression of Mx transcript in milt and egg without Poly I:C induction has not been reported earlier in Rohu. Mx proteins are found in mammals, birds, fish, and invertebrates indicating a probable role in protection against viral attack. Plant and Thune^25^ reported the presence of Mx production in ovary cells of channel catfish and an increase in Mx production after Poly I:C induction. Chinook salmon embryo (CHSE-214) cells showed an up-regulation of Mx expression after stimulation with IFN supernatants. Different isoforms of Mx have been reported in fish however not all isoforms of Mx are known for the expression of an antiviral protein. Thus, the exact nature of this Mx transcript expressed in egg, milt, and control-twitching stage without induction is unknown. It has been reported that the components of the immune system could be maternally inherited either as mRNA or as protein or both^57,58^. Huttenhuis (2006) have studied the maternally derived immune transcripts in *Cyprinus carpio* even in the unfertilized egg stage. The presence of proinflammatory cytokines like IL-1β and IL-6 has been studied in unfertilized egg stage up to a juvenile stage in *L. rohita* and a probable role of maternal transfer of immunity has been suggested by Dash^36^. An expression of Mx transcript in the early developmental stages of Rohu has not been reported earlier.

Mx and IRF3 genes which are known to be interferon stimulator genes and interferon regulator gene respectively presented significant up-regulation of expression in Zebrafish larvae on 11 dpf after Poly I:C induction^59^. The antiviral gene expression of TLR3 and TLR22 that recognize dsRNA^60^ are all expressed in lower levels in embryo compared to adults Zebrafish^61^. The antiviral gene expression in IFN-1, IFN-γ, Mx, TLR3, and MDA5, is also relatively lower in younger fish^59^. In this study, the expression of Mx transcript was considerably higher in egg, compared to milt and treated hatchling stage. Similar to the earlier reports of the transfer of maternal immunity like IgZ, IL-1β, IL-6 in *L. rohita*, Mx transcript is also maternally transferred in Rohu. As documented for other antiviral immune genes like TLR3, TLR22, MDA5, IFN-I, IFNγ, LGP2 expression of Mx is also very less in young fish compared to adult Rohu. From the study, it was found that the expression of Mx transcript in Rohu developmental stages was more pronounced in egg and twitching stages. Since the development of immune organs in carps is after 1month so the expression of Mx transcript in twitching stage might be maternally transferred from Rohu egg.

Immunoblot and RT-PCR have shown the presence of Rohu Mx protein in tissues of healthy individuals. Through Western blot of Poly I:C induced tissue samples Mx transcript was observed in all tissues of Rohu, but, more prominent in spleen, kidney, liver, muscle. In case of spleen and kidney, some putative bands were also seen apart from the major band at 72 kDa. After Poly I:C induction a putative Mx protein band of 71 kDa was identified in all organs of Atlantic halibut, but only faintly detected in organs of control fish. Approximately 71 kDa protein band was detected in IFN-induced HINAE cells that correspond with IFN-inducible Japanese Flounder Mx protein, which is predicted to be 70.7 kDa. The antiserum of Japanese flounder detected cross-reacting proteins of lower molecular weight. Similar lower molecular weight proteins were studied in other fishes and mammals^62^. In Poly I:C induced Atlantic salmon and rainbow trout more than one Mx gene has been reported, and the gene products have the same molecular mass^22,28^. Rohu Mx protein was detected in spleen, kidney, and liver even after 14 days of Poly I:C induction. Presence of Mx proteins 2 weeks after a single *in vivo* Poly I:C injection was also detected in organs of Atlantic salmon^23^ and different tissues of mice^2^. In control sample, Rohu Mx was detected in RT-PCR in all the tissues, the low levels of Mx expression in control might be due to constitutive expression of interferon production as has been reported in case of humans. It has been reported earlier that expression of Mx transcript in healthy fish of Japanese flounder, putative Mx mRNA expression was also observed in the kidney, spleen, intestine, brain, and gills of healthy individuals, with low levels of expression in leukocytes, liver, muscle and mucus of these animals^55^, Atlantic Salmon^23^ immunoblot detected the presence of Mx transcript in healthy individual. Mx was detected in Low levels of MxA mRNA expression was detected in mononuclear peripheral blood cells of healthy individuals, virus vaccination^55^. This lower level of Mx expression in healthy individuals can be attributed to the circulating IFN^63^.

Human, MxA is helpful against a broad range of viruses like orthomyxoviruses, bunyaviruses, picornaviruses paramyxoviruses, togaviruses, rhabdoviruses, reoviruses and Hepatitis B virus, a DNA virus with a genomic RNA intermediate^64^. How Mx protein prevents viral replication is still very unclear but in case of human MxA protein, it has been reported that MxA protein is localized in the cytoplasm of the IFN treated cells and prevents replication of the infected virus by preventing the oligomerization of viral capsids. Antiviral nature of Mx protein has been reported in higher vertebrates^54,62^. The exact nature of Rohu Mx is yet to be determined i.e., whether it has antiviral property or it is just an immune gene. Antiviral nature of Mx protein has been observed by Chen *et al*.^65^ in grouper cell lines that showed resistance to viral infection. It has been reported that rhabdoviruses infection has been prevented in a fish cell line transfected with Japanese flounder Mx protein^66^. Similarly, CHSE-214 cells expressing salmon Mx1 inhibited IPNV viral protein synthesis^67^. However, expression of Mx proteins in a Chinook salmon cell line does not interfere with accumulation of IHNV nucleoprotein^21^. Even Atlantic Salmon Mx protein does not prevent replication of infectious salmon anaemia virus (ISAV), an orthomyxovirus even though its replication is restricted in higher vertebrates.

Though our study highlighted the structure-function mechanism and mode of GTP binding to GTPase domain, there are still many questions, which need to be answered. Not all Mx proteins are antiviral in nature, as no antiviral activity has been demonstrated for avian Mx, and not for all Mx teleost Mx proteins. Conservation of Mx proteins amongst fish, avians and mammals show that they might have diverse roles in other activities like cellular transport, endocytosis thus indicating a possibility of a non-immune role. Mx proteins are homologous in nature to other non-immune GTPases that do not have immune activity. Again, the mode of action of each Mx varies for different viruses. Thus, the exact nature and function of Mx protein of Rohu need to be studied extensively to understand its role in antiviral mechanism in near future.

## Conclusion

The IFN-induced Mx proteins belonging to the evolutionary conserved dynamin-like large GTPases family (where GTPases activity directly proportional to antiviral activity) exhibits a wide spectrum antiviral activity against many viruses. Though they form dimers, tetramers, and oligomeric ring-like structures *in vitro*, the molecular mechanism of action remains to be completely understood in fishes. Therefore, to understand the molecular principle of Mx protein in Rohu, a combined approach involving both biotechnological and bioinformatics were used to investigate the dynamics and mode of GTP binding. The cDNA of rohu Mx protein was cloned and expressed in different tissues and a comparative sequence-structure analysis was performed with close structural homolog from mammals. Like the crystal structure human MxA, Mx protein of Rohu displayed the tripartite domain architecture comprised of GTPase binding domain (with dynamin signature and GTP-binding motif), central bundle signaling element (BSE) connected the amino-terminal GTPase domain and C-terminal domain with the Leucine zipper motifs architecture. Furthermore, Mx protein, which requires Mg^2+^ ion for GTPase activity, was found to bind the residues of dynamin signature in the GTPase domain, which perfectly corroborate with experimental findings with that of human. Molecular-mechanics based MM/PBSA binding analysis displayed that apolar solvation energy contributes significantly to the binding of GTP where van der Waals and electrostatic interaction were the major driving force behind the recognition mechanism. The results from the present study would be quite useful to understand the conformational changes caused upon GTP binding and antiviral mechanism in an enzymatically active form (multimeric assembly) of Mx protein in fishes.

## Materials and Methods

### Collection of samples and study design

Healthy Rohu 80 ± 10 g free of any (bacterial and viral infections) were maintained in 500 L aerated tanks in ICAR-Central Institute of Freshwater and Aquaculture, Kausalyaganga, Bhubaneswar, Odisha, India hatchery at an ambient temperature of 28 °C. Before starting the experiment, fishes were allowed to acclimatise to indoor conditions. The control group comprised of 40 Rohu fishes that were injected intraperitoneally with (I.P.) with 100 µl of phosphate buffered saline (PBS) and 40 Rohu fish were injected with 100 µl of the double-stranded synthetic RNA, Poly I:C (Sigma, USA) at a concentration of 2 mg/ml in PBS.

### Collection and treatment of Rohu eggs, hatchlings and fry

Stripping method was used for the collection of eggs from five gravid females. In the breeding season, milt was collected from five healthy males and both the egg and milt were preserved in liquid nitrogen. Fertilized eggs were transferred to hatchling tanks with sprinklers were water is maintained at 25 °C. Three different batches of one hundred eggs were collected including unfertilized and fertilized eggs. The RNA and protein was extracted using the TRIZOL method. The fertilized eggs were dipped in 15 mg/ml Poly I:C for 1 h. The treated fertilized eggs and the control-fertilized eggs were collected in hundreds in triplicates and preserved in liquid nitrogen for further use. Rohu twitchings were dissolved in 15 mg/ml of Poly I:C for 1 h and fifty number of treated twitching and fifty number of control twitching were collected in triplicates, preserved in liquid nitrogen. Rohu hatchlings were collected on 24, 48, 72 and 96 h and dipped in 15 mg/ml Poly I:C in 100 ml of water for 1 h. Hatchlings were in a group of 10 and in triplicates. Similarly, control hatchlings ten in number were collected in triplicates. Rohu fry was injected intramuscularly with 6 µl of Poly I:C (conc.1 mg/ml) in microneedle and Mx kinetics was observed for a period of 28 days. The fish samples for this study were collected in accordance with the ICAR-Central Institute of Freshwater Aquaculture (CIFA), Bhubaneswar, India ethical guidelines, and regulations. Further, the experimental protocols for this study were approved by the Institute Research Committee of ICAR-CIFA (Project Code: E-75).

### Poly I:C induction and RNA extraction

After Mx induction fish were anaesthetized 50 mg/l Ethyl-m-aminobenzoate methanesulphonate (benzocaine) prior to dissection. Aseptically, brain, kidney, liver, heart, spleen, intestine, muscle, gill and blood were collected from the fish on 2, 3, 5, 7 and 14 days post-injection. RNA was extracted from the collected tissues using Tri Reagent (Sigma, USA).

### cDNA synthesis

After RNA extraction, it was treated with DNaseI (Sigma, USA) to remove possible genomic DNA contamination. RNA quality was checked spectrophotometrically and 1 µg of RNA was reverse transcribed using cDNA synthesis kit (Invitrogen) following manufacturer protocol.

### PCR and cloning Mx cDNA fragment

Fast PCR software was used to design primer from *Ctenopharyngdon idella* and *Danio rerio* Mx to get a full-length sequence of Mx cDNA. Seven set of primer was designed to amplify different segments of the Mx gene (See Table 3). The amplified product was purified, using PCR purification kit (Gen Elute^TM^ PCR Clean-Up kit, Sigma, USA) and ligated into pGEMT Easy vector® (PROMEGA) plasmid vector). *E. coli* competent cells were used for transformation and through blue-white screening positive clones were selected. Plasmid PCR confirmed the presence of desired band then the plasmids were submitted for sequencing.

**Table 3:** Primer used in PCR reaction (F = Forward primer and R = Reverse primer).

Primer	5′-Primer seq	Target gene
2F241–261Mx	CGCTGCCTAGGGGAACAGGT	MxcDNA
3R713–734Mx	CCTTTGTCCACCAAGTCCGGT	MxcDNA
3F592–613Mx	GACAAGAAACCATCAGCTTGG	MxcDNA
4R1048–1068Mx	AGTAGGAACTCCAGCTCCCA	MxcDNA
4F1034–1055Mx	GGATCTTAGAGCACTGGGAGC	MxcDNA
5R1591–1612Mx	GCTGTAGAGACGGTCTTGGGA	MxcDNA
5F1598–1619Mx	ACCGTCTCTACAGCAGTCAGC	MxcDNA
6R1807–1828Mx	TCCAATCATGGCAAGCATTGC	MxcDNA
qPCR 6F1762–1784Mx	GTCCAGTACCACATGCTGGACC	MxcDNA
qPCR 7R1905–1927Mx	TTTGCCAGCACTCCTCAGGCGT	MxcDNA
Forward primer	TTCGAGCAGGAGATGGGCACTG	β-actin
Reverse primer	GCATCCTGTCAGCAATGCCA	β-actin
3′Gene Racer Primer	GCTGTCAACGATACGCTACGTAACG	
3′Gene Nested Primer	CGCTACGTAACGGCATGACAGTG	
5′Gene Racer Primer	GCACGAGGACACUGACAUGGACUGA	
5′Gene Nested Primer	GGACACTGACATGGACTGAAGGAGTA	

### Amplification and cloning of the full-length Rohu Mx gene

Sequencing of partial Rohu Mx gene 1784 bp was completed and 5′ and 3′ end terminal sequencing was performed by using RACE (Rapid Amplification of cDNA ends) through Invitrogen Generacer kit. Rohu spleen after 3^rd^ day post Poly I:C induction was used for RNA extraction, 5′RACE or “anchored” PCR method was done as per Frohman^64^. RNA was treated with CIP (Calf Intestinal Phosphatase), the dephosphorylated RNA was treated with TAP (Tobacco Acid Phosphatase) to remove cap from the complete mRNA. An RNA oligo was ligated at the 5′end of the mRNA which provides a binding site to the Gene Racer primers. For 5′end cDNA synthesis gene-specific primer was used for reverse transcription. First strand 3′end cDNA synthesis was done using oligodT anchored primer. Amplification was performed on an Applied Biosystems Veriti Thermal Cycler. The gene specific primer were designed from the 3′end was named as 6F (1762–1784) (5′ GTCCAGTACCACATGCTGGACC 3′) and 5′GSP as 3R(713–734) (5′CCTTTGTCCACCAAGTCCGGT 3′). The 3′ end of the gene was amplified using a gene-specific primer as the forward primer and the GeneRacer 3′primer to oligodT as the reverse primer. The cycling conditions were 94 °C for 2 min, 5 cycles at 94 °C for 15 sec, 60 °C for 30 sec, 68 °C for 45 sec, 30 cycles at 94 °C for 15 sec, 55 °C for 30 sec, 68 °C for 2 min and final extension at 68 °C for 10 min. The 5′end of the gene was amplified using a Generacer primer to the RNA oligo previously ligated in the cDNA synthesis process as forward primer and a gene specific to act as the reverse primer. The cycling parameters for the 5′end PCR were: 94 °C for 2 min, 35 cycles of 94 °C for 15 sec, 58 °C for 30 sec, 68 °C for 1 min, a final extension at 68 °C for 10 min. A nested PCR was done using GSP2 reverse primer and 5′end Generacer Nested primer, GSP2 forward, and Generacer Nested primer at 3′end. Products were visualized on a 1% agarose gel, purified and the purified product cloned into pGEMT vector and six clones were sequenced for 3′ and 5′ end products. From this sequence, subsequently, primers were designed at the start and stop codon to amplify the entire Rohu Mx gene sequence (See Table 1). The cDNA synthesized for RACE, was used in this PCR reaction. Amplification reaction was done in 25 µl of a reaction mixture containing 1 µl of cDNA, 1 unit of Jumpstart Taq DNA polymerase (Sigma, USA), 100 mM of dNTP mix each (Sigma, USA), 2.5 mM MgCl_2_ and 0.5 µM each primer. Cycling conditions were 94 °C 30 sec and 35 cycles of 94 °C 30 sec, 58 °C for 45 sec and 68 °C for 2 min and 68 °C extension for 10 min. Products were visualized in 1.2% agarose gel and custom sequenced to confirm the full length.

### RT-PCR

Quantification of Mx cDNA was carried out using SYBR Green fluorescent dye. The primers 6F1762–1784Mx (5′-gtccagtaccacatgctggacc-3′ and 7R1905–1927Mx (5′-tttgccagcactcctcaggcgt-3′) were used to amplify a 167 bp fragment of Rohu Mx cDNA. Quantitative real-time PCR (qRT-PCR) of the target Mx gene and β-actin (reference gene) were performed in Light Cycler®480 II-real time PCR detection system (Roche, Germany). PCR reaction were carried out in 10 µL reaction volume, containing 1 µL of cDNA, 0.5 µL of primer (25 nM each), 5 µL of 2X Light Cycler 480 SYBR Green I mastermix (Roche, Germany) and 3.5 µL of nuclease-free water. PCR amplifications were done in triplicates under the following conditions: initial denaturation at 95 °C for 10 min followed by 40 cycles of 95 °C/10 s, 55 °C/10 s and 72 °C/10 s. The PCR reactions were optimized using serial dilutions of cDNA, when efficiency was ~100%, 2^−ΔΔCT^ method for calculation of relative gene expression of the target gene Mx, with that of the reference gene, β-actin was done. The qRT-PCR product was run in 1.5% agarose gel to check single band amplification and the desired band length. Using the 2^−ΔΔCT^ method the data are represented as fold change in the gene expression, normalized to reference gene^68^. The data obtained from qRT-PCR analysis were expressed as a mean of three individual values ± standard error (SE), the significant difference between control and treated groups was analyzed statistically. The relative expression ratio (R) of the target gene was calculated based on the primer efficiencies (E) and the Ct deviations (ΔCt) of the investigated samples versus a calibrator, and expressed in comparison to the β-actin (reference gene).

### SDS PAGE

Protein was processed from the sediment tubes after RNA extraction by TriZOL method. DNA was precipitated adding 100% ethanol by centrifuging at 2000 × *g* for 5 min at 4 °C. The supernatant was discarded and washed three times with 0.3M Guanidine hydrochloride in 95% ethanol. Incubated at room temperature for 20 mins and then centrifuged at 7500 × g for 5 min at 4 °C. Protein pellet washed in 100% ethanol thrice and then incubated at room temperature for 20 min. Then the supernatant was discarded after centrifuging in7500 × *g* for 5 min at 4 °C. Tris-HCl 1:1 solution in 8M Urea and 1% SDS was added to the protein pellet and sonicated. A clear supernatant was obtained that was lyophilized and protein sample preserved in −80 °C. Samples were mixed with sample buffer containing 0.2M dithiotheritol (DTT) and 0.1% bromophenol blue, boiled for 2 mins and loaded in 10% SDS PAGE.

### Peptide synthesis

A peptide fragment was chosen from the amino acid sequence of Mx protein of Rohu (GenBank ID: KR349112) that was in consensus with other Mx protein sequence from *Cirrhinus mrigala* Mx (GenBank ID: KT371377), *Catla catla* Mx (GenBank ID: KP282448). This fragment was chosen to be in a region where the sequences were highly conserved. Care was taken to exclude the GTP binding region where Mx proteins have strong homologies with other GTP binding proteins. The peptide sequence was designed from Sigma that was 98% pure.

### Antiserum against Mx protein

The Synthesized peptide of 100 µl (about 100 µg of protein) was added to equal volume of PBS (pH 7.2) and 250 µl of Freund’s Complete Adjuvant (FCA). Similarly, emulsion of antigen with Freund’s incomplete adjuvant (FIA) was also prepared. The rabbit was intramuscularly injected into the hind leg with emulsion of FCA and FIA at a dose rate of 500 µl. Booster doses of immunization was given on 14^th^ and 28^th^ day with same dose of FIA. Blood from vein was collected by ear vein puncture on 42^nd^ days of immunization respectively. Serum was collected and preserved for further use^69^.

### Western blot

Four protein samples containing 10 µg of protein each was loaded on 10% SDS PAGE and electrophoresis was done in Bio-Rad electrophoresis cell. Proteins were transferred to a nitrocellulose membrane by dry blotting (90 min 100 mA) in Amersham Dry Blot Unit. All the staining procedure was followed according to protocol provided by Vector lab and reagents of Vectastain Elite ABC Kit were used.

### Statistical analysis

The mean values of these parameters were recorded over a period of 30 days and Duncan’s multiple range test (DMRT)^70^ were performed on the data (SAS version 9.2) to find the difference at 5% (P ≤ 0.05) level.

### Domain architecture and modeling of Mx protein

The nucleotide and protein sequence similarities were studied using the BLAST program of National Centre for Biotechnology Information (http://blast.ncbi.nlm.nih.gov/Blast.cgi). The protein domain and family of Mx protein was identified by Simple Modular Architecture Research Tool (SMART)^71^, and Pfam database search^72^. A scan of sequence in the PROSITE database aid in the prediction of the potential glycosylation sites^73^. Phylogenetic and molecular evolutionary analysis was conducted using the Neighbor-Joining method with 1000 bootstrap replications in MEGAv7 software package^74^. Theoretical modeling protocol employed in Modeller v9.19 was employed to predict the full-length three-dimensional (3D) architecture of Mx protein. Based on target-template alignment, raw 3D models were generated and subsequently refined. The refined models were then evaluated to test the structural integrity through various online model evaluation serves including PROCHECK^75^, ProSA-Web^76^, MolProbity^77^ and ProQ^78^.

### System preparation and molecular dynamics simulation

In this study, we employed coordinate transfer protocol to obtain starting structure of GTP by structural superimposition of GMPPCP-bound stalkless-MxA from Homo sapiens (PDB ID: 4P4S) with modeled Mx protein using PyMOL. We have truncated the modeled Mx protein and the N-terminal GTPase (G) domain that binds and hydrolyzes GTP (40–313 aa) was employed to get the starting structure GTPase-GTP staring structure. The parameters and procedure of MD were adopted from our previous studies^79,80^. To investigate the intrinsic dynamics stability, conformational flexibility and of the mode of GTP binding the GTPase domain and GTPase-GTP ligand systems were subjected to 100 ns molecular dynamics simulations in explicit water model using AMBER99Sb force field GROMACSv5.1^81^ package. Ante Chamber Python Parser interface (ACPYPE) (http://webapps.ccpn.ac.uk/acpype) was used to parameterize the required topologies, atomic type and charge of GTP. Before running the production run, both the systems were equilibrated by running 1000 ps of NVT (isothermal-isochoric) and NPT (isothermal-isobaric) ensemble. Production runs of 100 ns with an integration time step of 0.2 ps were performed at a constant temperature and pressure using the leapfrog algorithm. To constrain all bonds during the equilibration, LINCS algorithm was used while long-range ionic interactions were approximated with the particle-mesh Ewald algorithm. Trajectory analysis was stored at every 0.2 ps during the MD simulation time for post-dynamic analysis.

### Trajectory analysis

The utility toolkits of GROMACS were used to analyze MD trajectories produced during the 100 ns of the production run to determine stability parameters including backbone root mean square deviation (RSMD), the radius of gyration (Rg), C-α root mean square fluctuation (RMSF), and intermolecular hydrogen bond (H-Bonds) distribution for the complex system. The number of distinct H-bonds formed between specific amino acids residues and ligand atoms was determined to utilize the *gmx hbond* with the donor-acceptor set at a maximum of 0.35 nm. PyMOL was employed to scrutinize the intermolecular contacts, while, Xmgrace (Grace 5.1.21) was employed to plot the 2D graphs. Principal component analysis (PCA), was employed to probe conformational changes of proteins and protein-ligand system on the resultant MD trajectories using *gmx covar* and *gmx anaeig* utility toolkits^82–84^.

### Binding free energy calculation

The Molecular Mechanics/Poisson-Boltzmann Surface Area (MM/PBSA) method employed in *g_mmpbsa* was used to compute the binding free energy. A total of 500 snapshots were extracted from the last 30 ns (70–100 ns) an equal interval of time for estimation of binding free energy (ΔG_bind_) which can define by the following equation:

$${{\rm{\Delta }}{\rm{G}}}_{{\rm{bind}}}={{\rm{G}}}_{{\rm{Mx}}-{\rm{GTP}}{\rm{complex}}}-({{\rm{G}}}_{{\rm{Mx}}}+{{\rm{G}}}_{{\rm{GTP}}})$$

The G_Mx-GTP complex_, G_Mx_ and G_GTP_ represent the free energies of the Mx-GTP complex, Mx protein and GTP ligand, respectively. The methodology for binding energy estimation was adopted from previously published literature^85,86^.

## Supplementary information

Supplimentary Info
