# Supplementary material for: Molecular cloning, GTP recognition mechanism and tissue-specific expression profiling of myxovirus resistance (Mx) protein in Labeo rohita (Hamilton) after Poly I:C induction
Source: Sci Rep. 2019 Mar 8;9:3956. doi: 10.1038/s41598-019-40323-0 (PMC6408538; doi:10.1038/s41598-019-40323-0)
Supplement: Supplementary file 1 — Supplimentary Info [file 41598_2019_40323_MOESM1_ESM.docx]

**^±^Supplementary Information**

**Molecular cloning, GTP recognition mechanism and tissue-specific expression profiling of myxovirus resistance (Mx) protein in *Labeo rohita* (Hamilton) after Poly I:C induction**

Basanta Kumar Das^1,2^*, Pragyan Roy^1^, Ajaya Kumar Rout^2^, Deepak Ranjan Sahoo^1^, Soumya Prasad Panda^1^, Sushmita Pattanaik^1^, Budheswar Dehury^3,4^, Bijay Kumar Behera^2^, Sudhansu Sekhar Mishra^1^

^1^Fish Health Management Division, ICAR-Central Institute of Freshwater Aquaculture, Kausalyaganga, Bhubaneswar-751012, India

^2^Biotechnology Laboratory, ICAR-Central Inland Fisheries Research Institute, Barrackpore, Kolkata-700120, West Bengal, India

^3^Biomedical Informatics Centre, ICMR-Regional Medical Research Centre, Nalco Square, Chandrasekharpur, Bhubaneswar-751023, Odisha, India

^4^Department of Chemistry, Department of Chemistry, Technical University of Denmark, DK-2800 Kongens Lyngby, Denmark (Present Address)

^*^Corresponding Authors

Basanta Kumar Das

Email: basantakumard@gmail.com

Tel: +91-033-25921190 (O)

Fax: +91-033-25920388

R K Q E K K

358 ctaaaagacccagcagaaatagaaaatgctgtcttaaatgctcag

L K D P A E I E N A V L N A Q

403 atagcattggctggaaagggtgaagggatcagtcatgagatgatc

I A L A G K G E G I S H E M I

448 actctggagatccagtccagtgatgttcctgacctcactctcatt

T L E I Q S S D V P D L T L I

493 gacctgccaggcattgccagagttgccactggaaaccagccaatg

D L P G I A R V A T G N Q P M

538 gacattgagcaacaaataaaaagtctaattgaaacgttcattaaa

D I E Q Q I K S L I E T F I K

583 agacaagagaccatcagcttggttgtggtgcctgcaaacattgac

R Q E T I S L V VV P A N I D

628 atcgctaccactgaggctctgcggatggcatccaaagttgattca

I A T T E A L R M A S K V D S

673 acaggacaaagaactttgggtattctgactaagccagacttagtg

T G Q R T L G I L T K P D L V

718 gacaaaggcatggagcagacagtggtcagaacagtcaataatcaa

D K G M E Q T V V R T V N N Q

763 gtaataccactgaagaagggctacatgattgttaagtgcagaggc

V I P L K K G Y M I V K C R G

808 cagcaagacatcaatgagaaacttgatctggtcaaagctctggaa

Q Q D I N E K L D L V K A L E

853 aaagaaagacatttttttaatgaaaattctcattttaggtccctt

K E R H F F N E N S H F R S L

898 cttgaagatggaaaagctacaataccccttcttgcagaaagactc

L E D G K A T I P L L A E R L

943 acaaaagaactggttgaacacattactaaaacgctgccacagctg

T K E L V E H I T K T L P Q L

988 caaaaacaactcgagacgaaattagagaagacgtctgaggatctt

Q K Q L E T K L E K T S E D L

1033 aaagcactaggagatggagttcctcttgatgaaaatgagaagacc

K A L G D G V P L D E N E K T

1078 aattttctgattatgaaaattcgccagttcaatgacgcccttgaa

N F L I M K I R Q F N D A L E

1123 ggagtgaggagggcagaagaagatctaaaaaactcagatacaagg

G V R R A E E D L K N S D T R

1168 gtcttttccaaaatcagatgggaatttggaaaatggaaactcgcc

V F S K I R W E F G K W K L A

1213 ctggattccaaagcaattaagacggaggaaatcctcagagatgag

L D S K A I K T E E I L R D E

1258 gtggaggagtatgttaggactcgtagaggaaaggagcttcctgga

V E E Y V R T R R G K E L P G

1303 tttgtgaactacagaaccttcgagaacattgtcaggaaacatgtt

F V N Y R T F E N I V R K H V

1348 gaggagctagaggagcctgctttaaagatgctcagagaaatcaaa

E E L E E P A L K M L R E I K

1393 gaaattgttcacgcctgtgtggagcgtatagttaactctcatttt

E I V H A C V E R I V N S H F

1438 aatgccttctctcacctgctgagagctgcgaaagatccaattgaa

N A F S H L L R A A K D P I E

1483 gattttctcgatgagcagtttcagaaagctgaggggaaaatacat

D F L D E Q F Q K A E G K I H

1528 tctcagtttaagatggagaaaattgtctactcccaagaccgtctc

S Q F K M E K I V Y S Q D R L

1573 tacagcagtcagcttgaaactgtaaaacaaaatctaacagttctt

Y S S Q L E T V K Q N L T V L

1618 ggccaaaaggcgctcatgagtgcagatgtacgcgagatggcgcag

G Q K A L M S A D V R E M A Q

1663 catctcactgcctatttcacgattacctctgaccgactggctaac

H L T A Y F T I T S D R L A N

1708 caaattccactgattgtccagtaccacatgctggaccaatacatc

Q I P L I V Q Y H M L D Q Y I

1753 tctcagcttcagaatgcaatgcttgccatgattggaaggaacaac

S Q L Q N A M L A M I G R N N

1798 ccaggaatcctgctccaagaagacagtgctgttgagcgtaaaagg

P G I L L Q E D S A V E R K R

1843 aaagaactgaaagagagactgggacgcctgaggagtgctggcaaa

K E L K E R L G R L R S A G K

1888 gaactgtgtgcattctgcgaaagtaagcgggcgttagggcgttgg

E L C A F C E S K R A L G R W

1933 ctgtag

1938Cggtcacagatgttttccccacccccgcctaaggcgccgttacagtttgccattcaggacctgtcttgttgcacagtcatagggaagggagattctggtgcgggctttttggttatttgtgttgcaatattaataagatgggtaacgccagggttctcgcagtgacgactttgaacttttactgcccgggatttgataggattcattatactcagaattgggcccgacttcaagacgcgggaattcgattagacccggacgcaatccgagtcagagtcaagccagaagtgagacatgtgcctcctaaataaaaataaaaaatggtgaaaggctaaaaaaaaaaaaaaaaaaaaaaaaaa

**Fig. S1** Nucleotide and amino acid sequences of Rohu (*Labeo rohita*) Mx cDNA. Boxes indicate the tripartite GTP-binding motifs. Gray box at amino acid positions 58±67 indicates the location of the dynamin family signature motif. The polyadenylation signal is shown in bold and underlined.


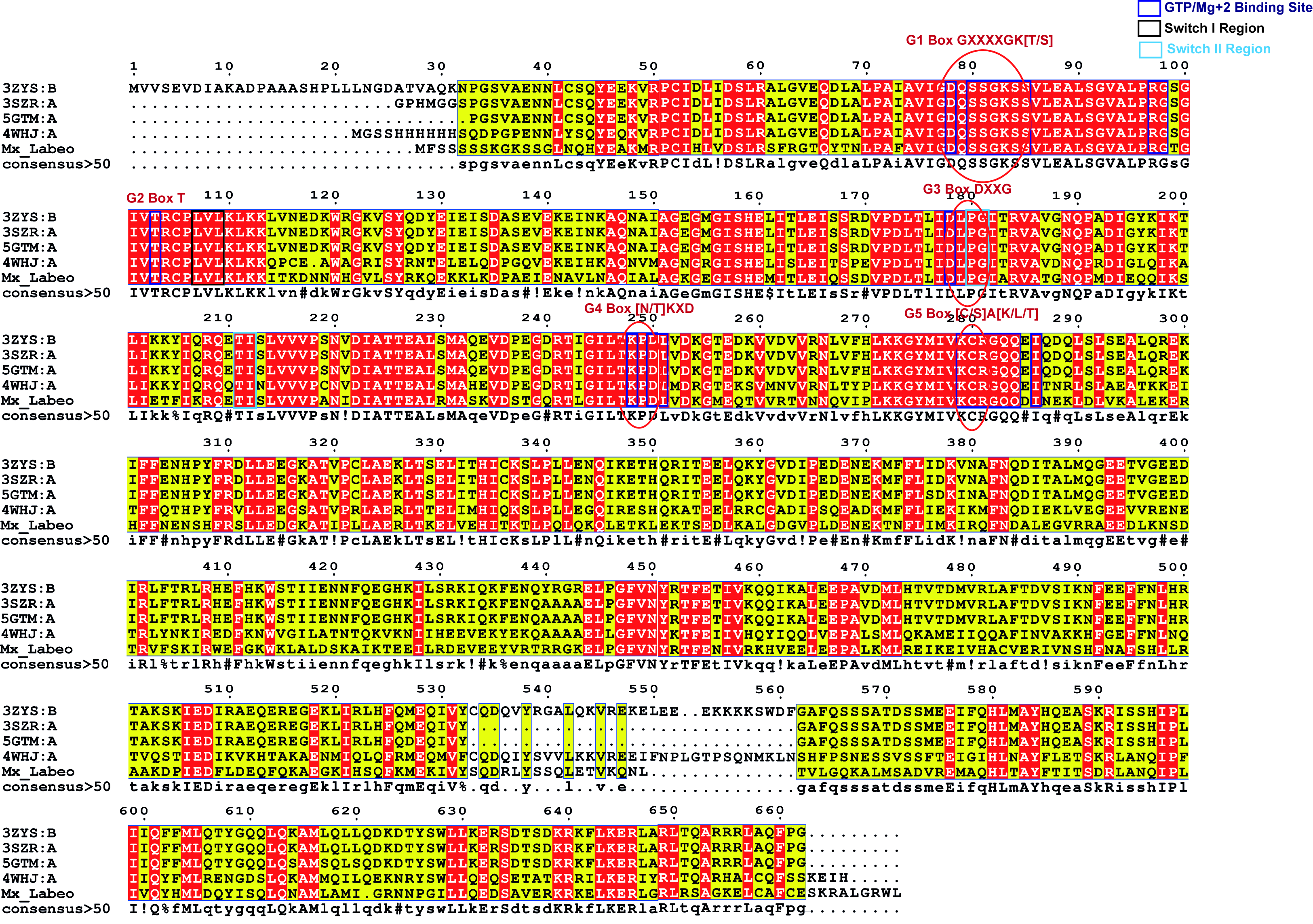


**Fig. S2** Multiple sequence alignment of Mx_*Labeo rohita* with its closest structural homologs. The alignment was performed using Multalin and rendered using ESPript3.0. Different important domains shared by Mx proteins have been labeled. The template include Hydrolase/GTP-Binding Protein (PDB ID: 3ZYS-B Chain), crystal structure of modified nucleotide-free human MxA (PDB ID: 3SZR-A), modified human MxA (PDB ID: 5GTM-A Chain), and Myxovirus Resistance Protein 2 (PDB ID: 4WHJ-A Chain).


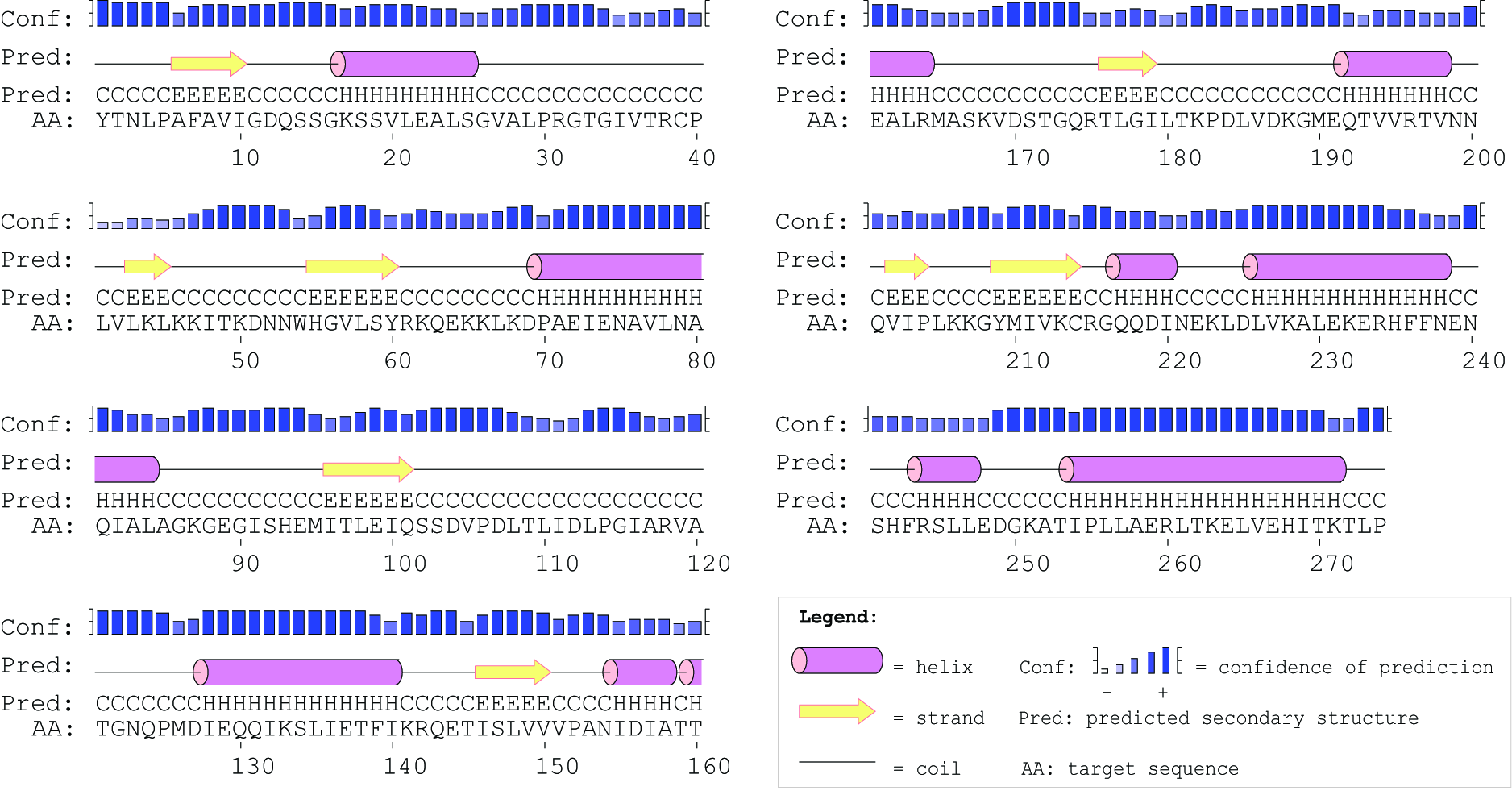


**Fig. S3** Secondary structure analysis of GTPase domain of Mx_*Labeo rohita* using PSIPRED server


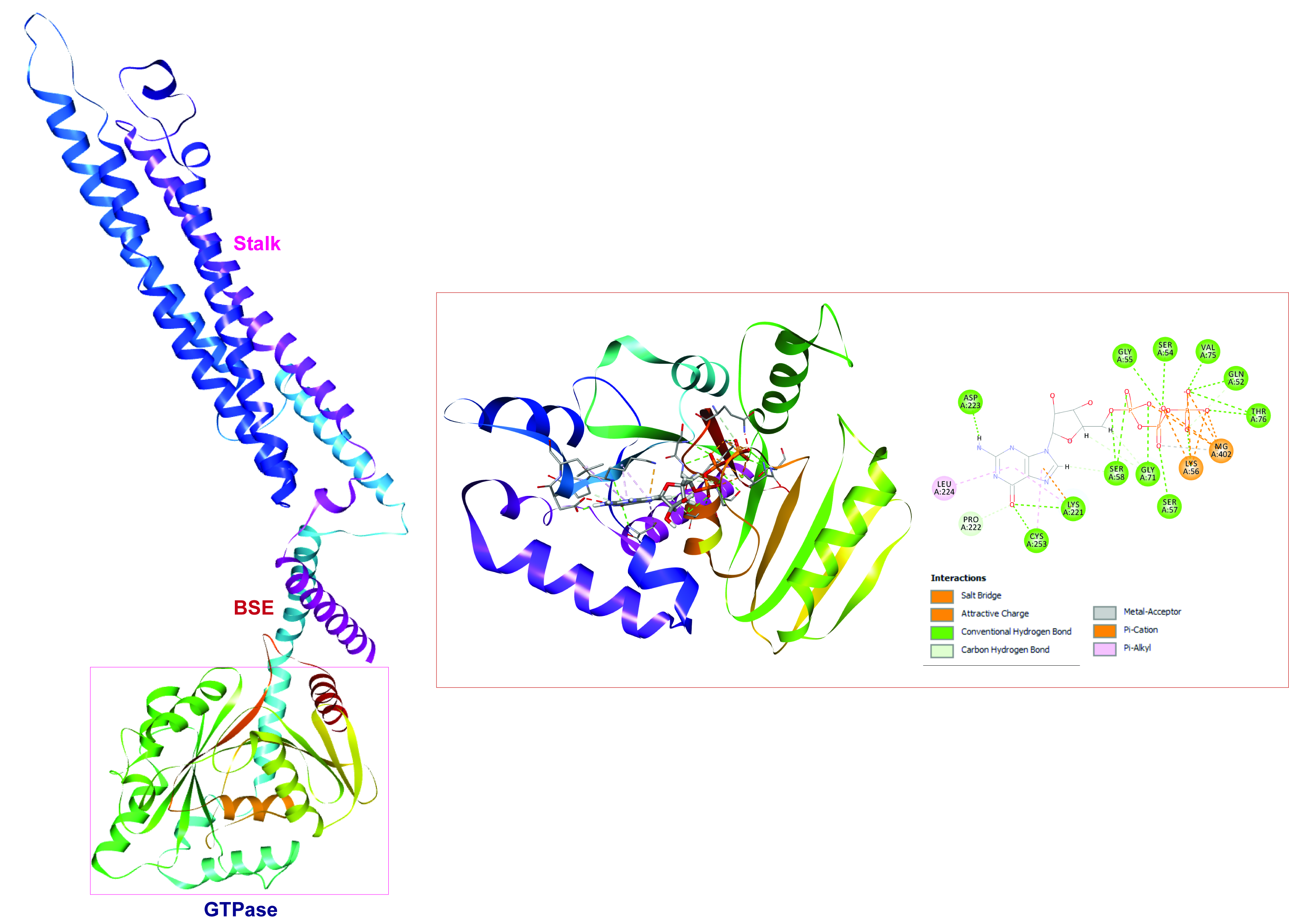


**Fig. S4** Solid ribbon-type representation of an Mx_*Labeo rohita* monomer, with three discrete domains (**A**). The protein ligand interaction of GTPase domain with GTP obtained using BIOVIA DSV (**B**). The different non-bonded contacts formed by GTP and Mg+2 ion have been displayed in the right panel.


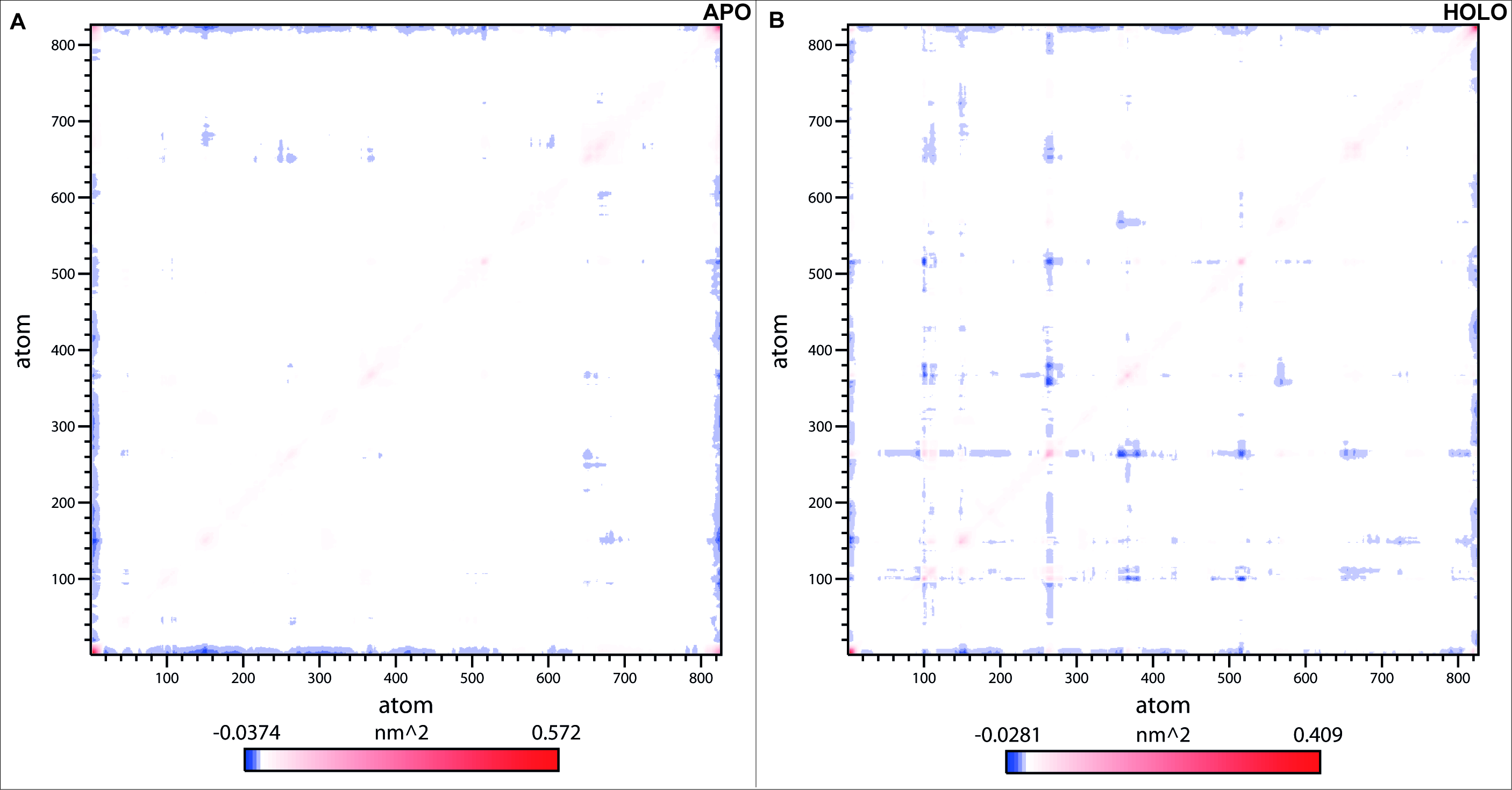


**Fig. S5** Principal component analysis of apo and holo conformers the GTPase domain of Mx_*Labeo rohita.* The cross-correlation matrices of the fluctuations of coordinates for Cα atoms around their mean positions during the 100 ns MD simulation.


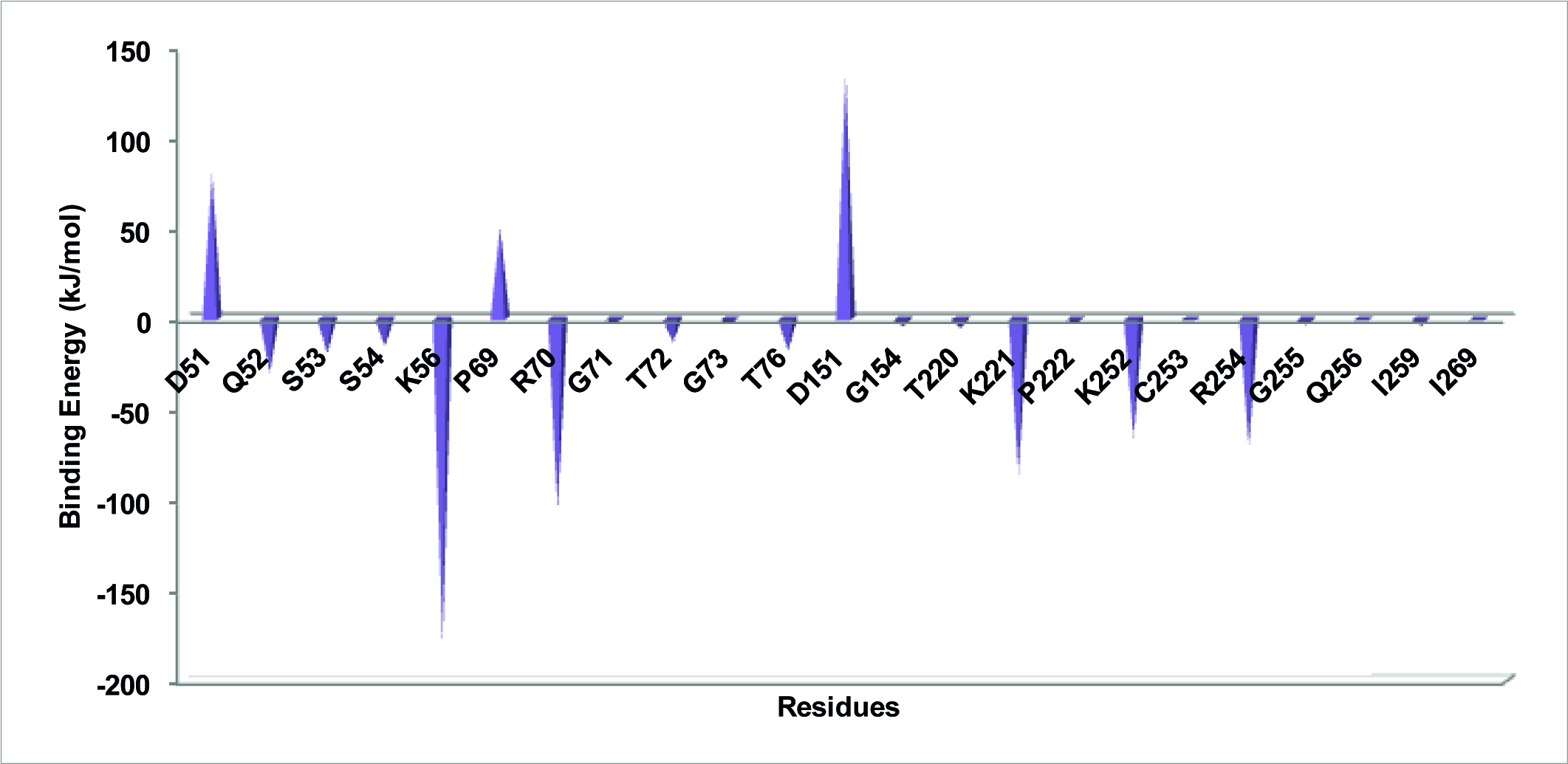


**Fig. S6** Per residue decomposition map displaying the energetic contribution of different amino acids to the overall MM/PBASA binding free energy of the GTPase-GTP complex. The Amino acids with a positive energy value impair the binding and vice versa. Free binding energy computation in 500 snapshots derived from the 70-100 ns MD simulation trajectory.

**Fig. S7** Ontogeny of Mx transcript in rohu in milt, egg, unfertilized eggs as well as in twitching stage (T) and hatchling stages(H24, H48, H72, H96).Treated twitching stages (TT) and treated hatchling stages (T H24, T 48, T H72, T H96). The fold change was calculated with respect to β-actin. The fold difference was calculated as 2^−ΔΔCq^, where ΔΔCq = (ΔCq sample −ΔCq calibrator) and ΔCq = (Cq value of Mx−Cq value of β-actin). Mean values bearing different superscripts are statistically different, P ≤ 0.05.


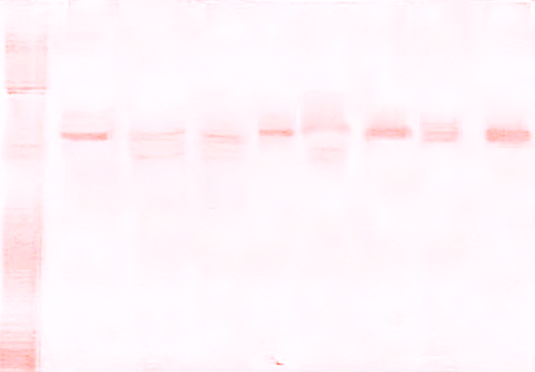


Mkr L 1 L 2 L 3 L 4 L 5 L 6 L 7 L 8

L2

72kDa

60kDa

45kDa

30kDa

92kDa

**Fig. S8** Immunostaining of the protein lysates of egg, milt, twitching, hatchling stages and Poly I:C treated twitching and hatchling stages Mkr, L 1 egg, L 2 milt, L 3 twitching stage, L 4 treated twitching stage, L 5 72h hatchling stage, L 6 72h treated hatchling stage, L 7 96h hatchling stage L 8 96h treated hatchling stage.

**Fig. S9** Expression of Mx transcript in fry in the entire experimental period of 28days. The fold change in Mx expression was normalized to housekeeping gene β-actin as measured by RT-PCR±SE, n=5. Samples with different symbols are statistically significant (P<0.05)


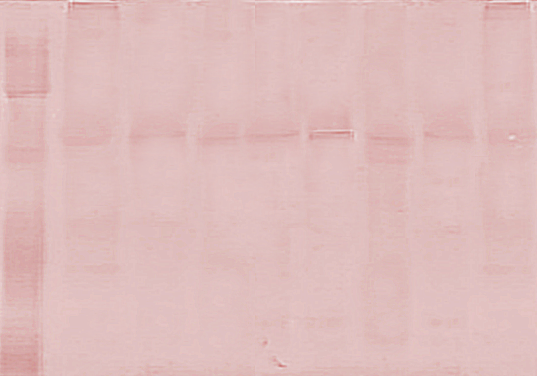


Mkr L1 L 2 L 3 L 4 L 5 L 6 L 7 L 8

30 kDa

45 kDa

60 kDa

92 kDa

72 kDa

**Fig. S10** Immunostaining of tissue lysates of rohu fry during the experimental period of 28 days Expression of Mx on day1 L1; day 2 L 2; day 3 L 3, day 5 L 4, day 7 L 5, day 11 L 6, day 21 L 7 and day 28 L8.

**Fig. S11** The GTP binding domain of Mx_*Labeo rohita* Protein was inferred using a Neighbor-Joining method. The percentage of replicate trees in which the associated taxa clustered together in the bootstrap test (1000 replicates) was displayed.

**
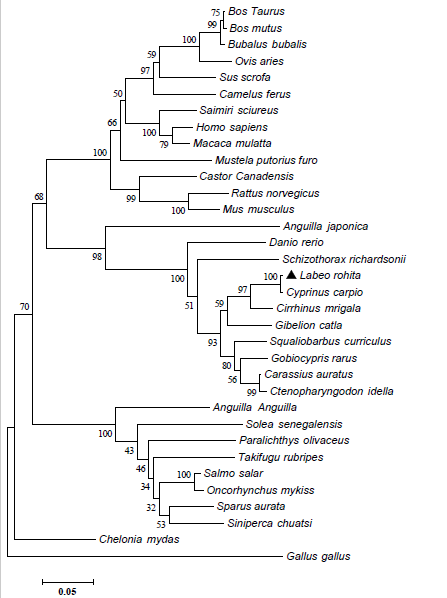
**

Table S1

Intermolecular contacts after MD simulation of GTPase domain of Mx protein with GTP (in presence of Mg^+2^)

| **Interaction** | **Distance** | **Category** | **Types** | **From** | **From chemistry** | **To** | **To Chemistry** |
| --- | --- | --- | --- | --- | --- | --- | --- |
| LYS56:NZ - GTP315:O1B | 2.76935 | Hydrogen Bond;Electrostatic | Salt Bridge | LYS56:NZ | H-Donor | GTP315:O1B | H-Acceptor |
| LYS56:NZ - GTP315:O1G | 2.99743 | Hydrogen Bond;Electrostatic | Salt Bridge | LYS56:NZ | H-Donor | GTP315:O1G | H-Acceptor |
| LYS56:NZ - GTP315:O1A | 5.50882 | Electrostatic | Attractive Charge | LYS56:NZ | Positive | GTP315:O1A | Negative |
| LYS56:NZ - GTP315:O3G | 4.79522 | Electrostatic | Attractive Charge | LYS56:NZ | Positive | GTP315:O3G | Negative |
| MG314:MG - ASP151:OD2 | 5.47029 | Electrostatic | Attractive Charge | MG314:MG | Positive | ASP151:OD2 | Negative |
| MG314:MG - GTP315:O1A | 3.54119 | Electrostatic | Attractive Charge | MG314:MG | Positive | GTP315:O1A | Negative |
| MG314:MG - GTP315:O1B | 2.12275 | Electrostatic;Other | Attractive Charge;  Metal-Acceptor | MG314:MG | Positive;  Metal | GTP315:O1B | Negative;  H-Acceptor |
| MG314:MG - GTP315:O1G | 1.87814 | Electrostatic | Attractive Charge | MG314:MG | Positive | GTP315:O1G | Negative |
| MG314:MG - GTP315:O3G | 1.88521 | Electrostatic | Attractive Charge | MG314:MG | Positive | GTP315:O3G | Negative |
| MG314:MG - ASP151:OD2 | 5.47029 | Electrostatic | Attractive Charge | MG314:MG | Positive | ASP151:OD2 | Negative |
| MG314:MG - GTP315:O1A | 3.54119 | Electrostatic | Attractive Charge | MG314:MG | Positive | GTP315:O1A | Negative |
| MG314:MG - GTP315:O1B | 2.12275 | Electrostatic;Other | Attractive Charge;Metal-Acceptor | MG314:MG | Positive;  Metal | GTP315:O1B | Negative;  H-Acceptor |
| MG314:MG - GTP315:O1G | 1.87814 | Electrostatic | Attractive Charge | MG314:MG | Positive | GTP315:O1G | Negative |
| MG314:MG - GTP315:O3G | 1.88521 | Electrostatic | Attractive Charge | MG314:MG | Positive | GTP315:O3G | Negative |
| GLN52:NE2 - GTP315:O3G | 2.94265 | Hydrogen Bond | Conventional Hydrogen Bond | GLN52:NE2 | H-Donor | GTP315:O3G | H-Acceptor |
| SER53:N - GTP315:O2B | 3.06126 | Hydrogen Bond | Conventional Hydrogen Bond | SER53:N | H-Donor | GTP315:O2B | H-Acceptor |
| SER57:N - GTP315:O1A | 2.81479 | Hydrogen Bond | Conventional Hydrogen Bond | SER57:N | H-Donor | GTP315:O1A | H-Acceptor |
| SER57:N - GTP315:O1B | 3.01111 | Hydrogen Bond | Conventional Hydrogen Bond | SER57:N | H-Donor | GTP315:O1B | H-Acceptor |
| SER57:OG - GTP315:O1B | 2.5807 | Hydrogen Bond | Conventional Hydrogen Bond | SER57:OG | H-Donor | GTP315:O1B | H-Acceptor |
| SER57:OG - GTP315:O1G | 2.72626 | Hydrogen Bond | Conventional Hydrogen Bond | SER57:OG | H-Donor | GTP315:O1G | H-Acceptor |
| THR76:OG1 - GTP315:O2G | 2.90568 | Hydrogen Bond | Conventional Hydrogen Bond | THR76:OG1 | H-Donor | GTP315:O2G | H-Acceptor |
| LYS221:N - GTP315:O6 | 3.06801 | Hydrogen Bond | Conventional Hydrogen Bond | LYS221:N | H-Donor | GTP315:O6 | H-Acceptor |
| CYS253:N - GTP315:O6 | 3.05148 | Hydrogen Bond | Conventional Hydrogen Bond | CYS253:N | H-Donor | GTP315:O6 | H-Acceptor |
| GTP315:O2' - ARG254:O | 3.21448 | Hydrogen Bond | Conventional Hydrogen Bond | GTP315:O2' | H-Donor | ARG254:O | H-Acceptor |
| SER53:CA - GTP315:O5' | 3.45352 | Hydrogen Bond | Carbon Hydrogen Bond | SER53:CA | H-Donor | GTP315:O5' | H-Acceptor |
| GTP315:C5' - SER58:OG | 3.7714 | Hydrogen Bond | Carbon Hydrogen Bond | GTP315:C5' | H-Donor | SER58:OG | H-Acceptor |
| GTP315:C2' - SER58:OG | 3.69107 | Hydrogen Bond | Carbon Hydrogen Bond | GTP315:C2' | H-Donor | SER58:OG | H-Acceptor |
| GTP315:C8 - SER58:OG | 3.03076 | Hydrogen Bond | Carbon Hydrogen Bond | GTP315:C8 | H-Donor | SER58:OG | H-Acceptor |
| MG314:MG - SER57:OG | 1.95133 | Other | Metal-Acceptor | MG314:MG | Metal | SER57:OG | H-Acceptor |
| MG314:MG - PRO69:O | 1.9767 | Other | Metal-Acceptor | MG314:MG | Metal | PRO69:O | H-Acceptor |
| MG314:MG - GTP315:O2A | 1.85298 | Other | Metal-Acceptor | MG314:MG | Metal | GTP315:O2A | H-Acceptor |
| MG314:MG - SER57:OG | 1.95133 | Other | Metal-Acceptor | MG314:MG | Metal | SER57:OG | H-Acceptor |
| MG314:MG - PRO69:O | 1.9767 | Other | Metal-Acceptor | MG314:MG | Metal | PRO69:O | H-Acceptor |
| MG314:MG - GTP315:O2A | 1.85298 | Other | Metal-Acceptor | MG314:MG | Metal | GTP315:O2A | H-Acceptor |
| LYS221:NZ - GTP315 | 4.79919 | Electrostatic | Pi-Cation | LYS221:NZ | Positive | GTP315 | Pi-Orbitals |
| GTP315 - LYS221 | 3.86345 | Hydrophobic | Pi-Alkyl | GTP315 | Pi-Orbitals | LYS221 | Alkyl |
| GTP315 - CYS253 | 5.36101 | Hydrophobic | Pi-Alkyl | GTP315 | Pi-Orbitals | CYS253 | Alkyl |
| GTP315 - LYS221 | 4.45198 | Hydrophobic | Pi-Alkyl | GTP315 | Pi-Orbitals | LYS221 | Alkyl |
| GTP315 - CYS253 | 4.70878 | Hydrophobic | Pi-Alkyl | GTP315 | Pi-Orbitals | CYS253 | Alkyl |

**Table S2**

A comparative analysis of the Mx protein sequences with Rohu Mx sequence

| **Organism** | **Type** | **Query coverage** | **Identity** | **Accession No** |
| --- | --- | --- | --- | --- |
| *Catla catla* | Mx protein | 91 | 92 | KP282448.1 |
| *Cirrhinus mrigala* | Mx protein | 90 | 92 | KP033198.1 |
| *Ctenopharyngodon idella* | Mx protein | 96 | 82 | AY395698.1 |
| *Squaliobarbus curiculus* | Mx protein | 96 | 81 | KC249972.1 |
| *Gobiocypris rarus* |  | 98 | 80 | EF095273.1 |
| *Carassius auratus* |  | 96 | 80 | AY303812.1 |
| *Danio rerio* | MxE | 97 | 76 | XM_005167664.2 |
| *Homo sapiens* | Mx1 | 92 | 51 | XM_005260978.3 |
